# Bioactive Phenolic Compounds from *Peperomia obtusifolia*

**DOI:** 10.3390/molecules27144363

**Published:** 2022-07-07

**Authors:** Ismail Ware, Katrin Franke, Hidayat Hussain, Ibrahim Morgan, Robert Rennert, Ludger A. Wessjohann

**Affiliations:** 1 Department of Bioorganic Chemistry, Leibniz Institute of Plant Biochemistry, D-06120 Halle, Germany; ismailbin.ware@ipb-halle.de (I.W.); hidayat.hussain@ipb-halle.de (H.H.); ibrahim.morgan@ipb-halle.de (I.M.); robert.rennert@ipb-halle.de (R.R.); 2 Institute of Bioproduct Development, Universiti Teknologi Malaysia, Johor Bahru 81310, Malaysia

**Keywords:** Piperaceae, *Peperomia obtusifolia*, isolation, cytotoxicity, anticancer, antibacterial

## Abstract

*Peperomia obtusifolia* (L.) A. Dietr., native to Middle America, is an ornamental plant also traditionally used for its mild antimicrobial properties. Chemical investigation on the leaves of *P. obtusifolia* resulted in the isolation of two previously undescribed compounds, named peperomic ester (**1**) and peperoside (**2**), together with five known compounds, viz. *N*-[2-(3,4-dihydroxyphenyl)ethyl]-3,4-dihydroxybenzamide (**3**), becatamide (**4**), peperobtusin A (**5**), peperomin B (**6**), and arabinothalictoside (**7**). The structures of these compounds were elucidated by 1D and 2D NMR techniques and HREIMS analyses. Compounds **1**–**7** were evaluated for their anthelmintic (against *Caenorhabditis elegans*), antifungal (against *Botrytis cinerea, Septoria tritici and Phytophthora infestans*), antibacterial (against *Bacillus subtilis* and *Aliivibrio fischeri*), and antiproliferative (against PC-3 and HT-29 human cancer cell lines) activities. The known peperobtusin A (**5**) was the most active compound against the PC-3 cancer cell line with IC_50_ values of 25.6 µM and 36.0 µM in MTT and CV assays, respectively. This compound also induced 90% inhibition of bacterial growth of the Gram-positive *B. subtilis* at a concentration of 100 µM. In addition, compound **3** showed anti-oomycotic activity against *P. infestans* with an inhibition value of 56% by using a concentration of 125 µM. However, no anthelmintic activity was observed.

## 1. Introduction

*Peperomia* is one of the two major genera of the Piperaceae family, with approximately 1700 species common in almost all tropical areas [1]. Plants of the genus *Peperomia* possess numerous uses as traditional medicine to treat asthma, gastric ulcers, bacterial infection, pain, and inflammation [2]. *Peperomia* species produce several classes of secondary metabolites such as flavonoids, secolignans, prenylated phenolic substances, chromenes, polyketides [3], lignans, tetrahydrofurans, terpenoids, meroterpenoids, and amides [1,4,5]. Some compounds from *P. pellucida*, *P. doclouxii*, *P. dindygulensis*, and *P. blanda* were reported to exhibit cytotoxic effects [2,6,7,8].

*Peperomia obtusifolia* (L.) A.Dietr. is widely distributed from Mexico to the northern part of South America. It is widely known as *baby rubber* or the *paragua* plant. In spite of its dominating decorative use, some communities in Central America use this plant to treat insect and snake bites and also as a skin cleanser [9]. Previous studies on *P. obtusifolia* resulted in the isolation of chromanes, flavonoids, lignans, and other secondary metabolites [10,11,12,13,14]. Several biological activities were described for compounds isolated from this species, such as trypanocidal [11], anti-inflammatory [15], and antibacterial activities [14]. In the present study, we report the isolation, structure elucidation, and biological effects of two previously undescribed compounds (**1** and **2**), along with five known compounds (**3**–**7**) from the methanolic leaf extract of *P. obtusifolia*. 

## 2. Results and Discussion

**Figure 1:**
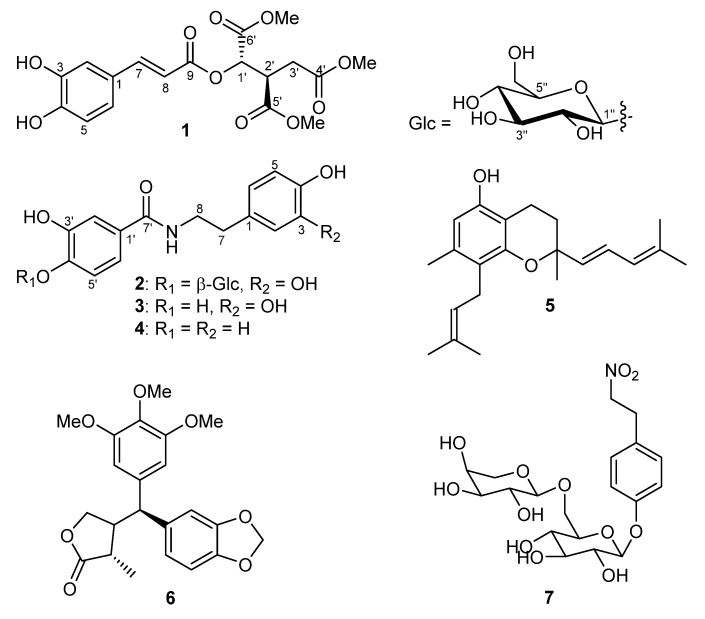
Chemical structures of compounds **1**–**7**.

The dried leaves of *P. obtusifolia* were extracted with 80% aqueous methanol. The crude extract was suspended in water and successively fractioned by liquid−liquid partition between water and *n*-hexane, and water and ethyl acetate. The constituents were purified by preparative HPLC and repeated column chromatography on silica gel, Sephadex LH20, and Diaion HP20, which yielded two previously undescribed compounds (**1** and **2**), as well as two amide derivatives (**3** and **4**), a chromane (**5**), a secolignan (**6**), and an unusual phenolic nitroalkyl diglycoside (**7**) (Figure 1). The chemical structures of **1** and **2** were elucidated by detailed spectroscopic techniques.

**Figure 2:**
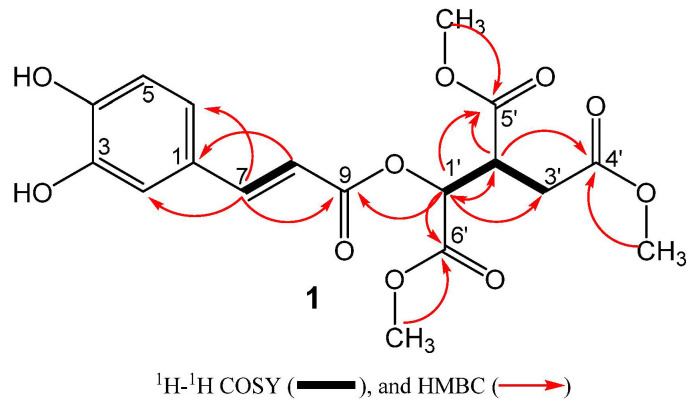
Key ^1^H-^1^H COSY and HMBC correlation of **1**.

Compound **1** was obtained as a brownish liquid from the aqueous fraction. The HR-ESI-MS measurements of the deprotonated molecular ion at *m*/*z* 395.0975 [M − H]^−^ indicated the molecular formula C_18_H_20_O_10_. The IR spectrum revealed the presence of hydroxyl (3402 cm^−1^) and carbonyl groups (1715 cm^−1^). Inspection of the ^1^H NMR data (Table 1) exhibited characteristic signals of an ABX aromatic system at δ 7.06 (1H, d, *J* = 2.1 Hz, H-2), 6.79 (1H, d, *J* = 8.2 Hz, H-5), and 6.97 (1H, d, *J* = 8.2, 2.1 Hz, H-6) and of one *E*-configured double bond [7.59 (1H, d, *J* = 15.8 Hz, H-7) at 6.29 (1H, d, *J* = 15.8 Hz, H-8)]. In addition, ^1^H NMR data also showed the presence of two saturated methine groups [δ 5.46 (1H, d, *J* = 3.9 Hz, H-1′) at 3.62 (1H, ddd, *J* = 9.0, 5.5, 3.9 Hz, H-2′), and one methylene group [δ 2.85 (1H, dd, *J* = 17.2, 9.0 Hz, H-3′a) at 2.66 (1H, d, *J* = 17.2, 5.5 Hz, H-3′b)] along with three methoxy groups at δ 3.68, 3.73, and 3.77. The ^13^C data (Table 1) exhibited one sp^2^ quaternary carbon [δ_C_ 127.4 (C-1)], two oxygenated sp^2^ tertiary carbons [δ_C_ 146.8 (C-3), 150.0 (C-4)], and four carbonyl groups [δ_C_ 167.5 (C-9), 173.2 (C-4′), 170.2 (C-5′), 172.1 (C-6′)]. The scaffold of compound **1** was established by an HMBC experiment, showing long-range correlations from H-7 to C-1, C-2, C-6, C-8, and C-9, indicating that the aromatic ring and the carbonyl group C-9 were linked to C-7 in the form of a caffeic acid moiety. In addition, COSY correlations of H-1′/H-2′, H-2′/H-1′/H-3′a/H-3′b, H-3′a/H-2′, and H-3b/H-2′, together with HMBC correlations of the three methoxy groups to their respective carbonyls (C-4’, C-5′, and C-6′) and HMBC correlations of H-1′ and C-2′, C-3′, C-5′, C-6′ and of H-2′ and C-1′, C-3′, C-4′, C-5′, and C-6′ suggested the presence of an isocitrate core. The C-1′ hydroxyl of the isocitrate unit is attached to the caffeic acid moiety at C-9 indicated by the long-range correlation from H-1′ (δ_H_ 5.46) to C-9 (δ_C_ 167.5) (Figure 2). The methylated isocitrate moiety of **1** is similar to those found in cryptoporic acids isolated from *Cryptoporus sinensis* [16] and *Cryptoporus volvatus* [17,18], which are also conjugates of isocitrate. Since compound **1** was also detected in an ethanolic crude extract (Figure S9), the methylation is not a processing artifact. The absolute configuration of C-1′ and C-2′ of the isocitrate group was suggested to be (1′*S*,2′*R*) by comparison of its optical rotation sign (negative optical rotation value) with published compounds having a (1′*R*,2′*S)* absolute configuration and opposite-in-sign specific rotation (positive optical rotation value) [16,19]. Thus, the methylated isocitrate moiety is in accordance with an L-threo-isocitric acid core ((1*S*,2*R*)-1-hydroxypropane-1,2,3-tricarboxylic acid). Based on the above-mentioned evidence, the structure of **1** was elucidated as trimethyl (1*S*,2*R*)-1-(((*E*)-3-(3,4-dihydroxyphenyl)acryloyl)oxy)propane-1,2,3-tricarboxylate and given the trivial name peperomic ester.

**Figure 3:**
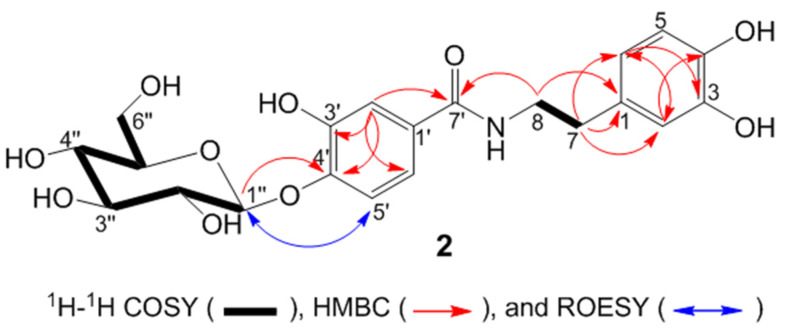
Key ^1^H-^1^H COSY, HMBC, and ROESY correlation of **2**.

Compound **2** (Figure 3) was obtained as a white amorphous powder from the ethyl acetate fraction. The HR-ESI-MS measurements of the deprotonated molecular ion at *m/z* 450.1379 [M − H]^−^ indicated the molecular formula C_21_H_25_NO_10_. The IR spectrum displayed absorption for hydroxyl (3250 cm^−1^) and carbonyl groups (1607 cm^−1^). The NMR data of compound **2** (Table 2, Figure 3) show the presence of an *N*-[2-(3,4-dihydroxyphenyl) ethyl]-3,4-dihydroxybenzamide moiety [20]. The structure of the aglycon part is the same as in compound **3**.

In addition, NMR data indicate the presence of an anomeric carbon [(δ_H_ 4.79 (1H, d, *J* = 7.5 Hz); δ_C_ 105.1] together with four methine carbon signals between 71.3 and 78.5 ppm, which is typical for glucose. The coupling constant value of the sugar anomeric proton (*J* = 7.5 Hz) revealed the *β*-configuration of the glucosyl moiety. The linkage of C-1″ from the sugar moiety to 4′OH of the 3,4-dihydroxybenzoate moiety was established based on the HMBC long-range correlation from H-1″ (δ_H_ 4.79) to C-4′ (δ_C_ 150.1) (Figure 3). This conclusion is supported by the ROESY correlation of H-1″ (δ_H_ 4.79) with H-5’ (δ_H_ 7.24). From the above spectroscopic data, the structure of compound **2** was established as *N*-[2-(3,4-dihydroxyphenyl)ethyl]-3,4-dihydroxybenzamide 4′-*O*-*β*-d-glucoside, given the trivial name peperoside.

The phytochemical investigation of the *P. obtusifolia* (L.) A.Dietr. leaves allowed the isolation of five other known compounds, *N*-[2-(3,4-dihydroxyphenyl)ethyl]-3,4-dihydroxybenzamide (**3**) [20], becatamide (also known as houttuynamide A, **4**) [21], peperobtusin A (**5**) [10], peperomin B (**6**) **[22]**, and arabinothalictoside (**7**) **[23]**. These compounds were identified by comparing their MS and NMR spectral data with published data, which agree with the data reported for the respective compounds. Among the known constituents, compound **5** was already reported from *P. obtusifolia*, while compounds **3**, **4**, and **6** were isolated from other *Peperomia* species such as *P. duclouxii* [7], *P. tetraphylla* [24], and *P. japonica* [22], respectively. Compound **7** is an unusual nitro natural product which was isolated for the first time from the Japanese plant *Sagittaria trifolia* [25] and later reported in *Aristolochia fordiana* [26], which belong to Piperales and *Annona squamosa* [23]. 

*Peperomia* species constituents have previously been reported to exhibit a variety of biological activities, either as extracts or pure compounds [2,27,28,29]. Thus, the compounds isolated from this species were evaluated for their anthelmintic, antifungal, antibacterial, and anticancer properties. These biological examinations were conducted by using established non-pathogenic model organisms and human cancer cell lines (all BSL-1) in fast-screening assays (Table 3 and Table 4). 

The anthelmintic activity was evaluated against *Caenorhabditis elegans*, however, the compounds did not significantly kill the nematodes even when applying a high concentration of 200 µM. Moreover, most of the compounds tested for antifungal effects against the phytopathogens *B. cinerea, S. tritici*, and *P. infestans* were considered as inactive since the inhibition of fungal growth at a concentration of 125 µM was less than 50%. An exception was *N*-[2-(3,4-dihydroxyphenyl)ethyl]-3,4-dihydroxybenzamide (**3**), which displayed interesting anti-oomycotic activity against *P. infestans* with an inhibition value of 56% at the same concentration. For the antibacterial assays, the compounds were tested in concentrations of 1 and 100 µM. None of the compounds inhibited the Gram-negative bacterium *A. fischeri* even at the highest test concentration of 100 µM. However, against the Gram-positive *B. subtilis*, peperobtusin A (**5**) induced a 90% inhibition of bacterial growth when treating at a concentration of 100 µM, prompting further investigations against human pathogens. Prenylated benzopyrans seem to be the active principle of *Peperomia* against Gram-positive bacteria, including resistant *Staphylococcus aureus* and *Enterococcus faecalis* strains [14]. Since all other isolated compounds induced less than 50% inhibition of bacterial growth when applying a concentration of 100 µM (Table 3), their half-maximal inhibitory concentration (IC_50_) values must be estimated to be greater than 100 µM.

Furthermore, the compounds **1**–**7** were screened for their potential antiproliferative or cytotoxic activity against two human cancer lines, namely prostate adenocarcinoma cells (PC-3) and colorectal adenocarcinoma cells (HT-29). The compounds’ effect on the metabolic cancer cell viability was determined by conducting an MTT assay, general cytotoxic effects were determined by using a CV assay, both after 48 h cancer cell treatment with the compounds under investigation. A very potent permeabilizer of cell membranes, digitonin (125 µM), was used as a positive control, compromising the cells to the point of 0% of cell viability after 48 h. The compounds were tested at two different concentrations, namely 10 nM and 10 µM. 

As shown in Table 4, most of the compounds exhibited no significant antiproliferative and cytotoxic effects on PC-3 and HT-29 cancer cells, with the exception of peperobtusin A (**5**), which strongly inhibited the viability and growth of PC-3 cells in an MTT and CV fast-screening assay after treating the cells with 10 µM of the compound for 48 h. Under the same fast-screening assay conditions, a moderate activity of compound **5** against the HT-29 colorectal cancer cell line was also observed.

**Figure 4:**
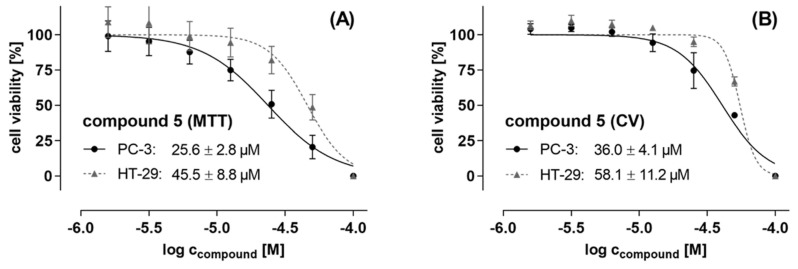
Effect of peperobtusin A (**5**) on (**A**) the metabolic cell viability of prostate PC-3 cancer cells and colon HT-29 cancer cells, as determined by MTT assay after 48 hours of cell treatment. (**B**) General cytotoxic and antiproliferative effect of **5** on both cancer cell lines under comparable treatment conditions, as determined by using crystal violet (CV) assay. Data represent biological duplicates, each comprising technical quadruplicates. IC_50_ curves were analyzed and drawn using SigmaPlot and GraphPad Prism software, respectively. IC_50_ values are given as mean ± SD.

Based on its antiproliferative and cytotoxic effects, as determined with a 10 µM concentration in the initial fast-screening assay, compound **5** was subjected to further evaluations of its IC_50_ values in both cancer cell lines. The cells were treated with increasing concentrations of up to 100 µM of compound **5** for 48 h, followed by an MTT and CV assay read-out, data analyses, and calculation of IC_50_ values. The IC_50_ values of compound **5** determined by MTT assay, indicating the metabolic viability of the cancer cells, were 25.6 ± 2.8 µM and 45.5 ± 8.8 µM in PC-3 and HT-29 cells, respectively (see Figure 4A). The CV assay, reflecting general cytotoxic and antiproliferative impacts, showed similar IC_50_ values of 36.0 ± 4.1 µM and 58.1 ± 11.2 µM for PC-3 and HT-29 cells, respectively (Figure 4B). However, given the result of the preliminary fast-screening (fixed concentration 10 µM) of compound **5**, which estimated an IC_50_ value of less than 10 µM, the lower efficacy at the above actual calculated IC_50_ values based on the dose-response curves was unexpected, especially in the case of PC-3 cells. However, the IC_50_ values were reproduced in three different assay formats, each with 2-3 biological replicates, namely MTT, CV, and resazurin assays (data not shown), which revealed an IC_50_ range of 25–45 µM. This implies that fast-screening with a single concentration and a single biological replicate, although with technical quadruplicates, is only a very rough, initial estimation of the compounds’ activity. 

Peperobtusin A (**5**) is a prenylated benzopyran with a chiral center at C-2. It was obtained with an optical activity of ${\left[\alpha \right]}_{\text{D}}^{20}$ 0 (*c* 0.28, CHCl_3_), implying a racemic mixture of the molecule. This result corroborated previously published data [11,30]. The compound was isolated before from the same species [10,11,14] and other *Peperomia* species, such as *P. tetraphylla* [31] and *P. clusiifolia* [32]. Although there are a few existing studies addressing the biological activities of peperobtusin A (**5**), this study is the first report related to the antiproliferative and cytotoxic effect against PC-3 (prostate) and HT-29 (colon) cancer cell lines. Previous investigations indicated the antitrypanosomal and antitumor potential of peperobtusin A. Regarding the antitrypanosomal activity, the compound with an IC_50_ value of 3.1 µM was almost three times more active than the positive control benznidazole [11]. Furthermore, Da Silva Mota and collaborators also evaluated its cytotoxicity in mammalian cells (murine peritoneal macrophages) and found the compound to not be toxic at the concentration level of its trypanocidal activity [11]. A recent study by Shi et al [31] revealed that peperobtusin A (**5**) had antiproliferation effects against the human lymphoma cell line U937 with an IC_50_ value of 63.26 µM at 24 h, however, exerted only minor effects on other human cancer cell lines such as human melanoma (A375), human bladder carcinoma (T24), and human breast epithelial cells (HBL-100). According to Shi et al. [31], the intracellular ROS formation and the activation of caspases and P38 MAPK played significant roles in the apoptosis induced by peperobtusin A. Therefore, in future investigations, the mode of action of peperobtusin A (**5**) in PC3 and HT29 cell lines should be addressed in detail and expanded; toxicological investigations should be performed to validate the safety of this compound before developing it as trypanocide.

## 3. Materials and Methods

### 3.1. General Methods

The following instruments were used to obtain physical and spectroscopic data: column chromatography was performed on silica gel (400–630 mesh, Merck, Darmstadt, Germany), Sephadex LH-20 (Fluka, Steinheim, Germany), and Diaion HP20 (Supelco, Bellefonte, PA, USA). Fractions and substances were monitored by TLC. TLC was conducted on precoated Kieselgel 60 F 254 plates (Merck, Darmstadt, Germany) and the spots were detected either by examining the plates under a UV lamp at 254 and 366 nm or by treating the plates with vanillin or natural product reagents. UV spectra were recorded on a Jasco V-770 UV-Vis/NIR spectrophotometer (Jasco, Pfungstadt, Germany), meanwhile specific rotation was measured with a Jasco P-2000 digital polarimeter (Jasco, Pfungstadt, Germany). The IR (ATR) spectra were carried out in MeOH using a Thermo Nicolet 5700 FT-IR spectrometer (Thermo Nicolet Analytical Instruments, Madison, WI, USA).

NMR spectra were obtained with an Agilent DD2 400 system at +25 °C (Varian, Palo Alto, CA, USA) using a 5 mm inverse detection cryoprobe. The compounds were dissolved in CD_3_OD (99.8% D) or CDCl_3_ (99.8% D) and the spectra were recorded at 399.915 MHz (^1^H) and 100.569 MHz (^13^C). One-dimensional (^1^H and ^13^C) and two-dimensional (^1^H,^13^C HSQC, ^1^H,^13^C HMBC, ^1^H,^1^H COSY, and ^1^H,^1^H ROESY) spectra were measured using standard CHEMPACK 8.1 pulse sequences implemented in the Varian VNMRJ 4.2 spectrometer software (Varian, Palo Alto, CA, USA). ^1^H chemical shifts are referenced to internal TMS (^1^H δ = 0 ppm), while ^13^C chemical shifts are referenced to CD_3_OD (^13^C δ = 49 ppm) or CDCl_3_ (^13^C δ = 77.0 ppm). 

The semi-preparative HPLC was performed on a Shimadzu prominence system (Kyoto, Japan) equipped with LabSolutions software, LC-20AT pump, SPD-M20A diode array detector, SIL-20A auto sampler, and FRC-10A fraction collector unit. Chromatographic separation was carried out using a YMC Pack C18 column (5 µm, 120 Å, 150 mm × 10 mm I.D, YMC, USA) using H_2_O (A) and CH_3_CN (B) as eluents at a flow rate of 2.2 mL min^−1^. 

The high-resolution mass spectra in both positive and negative ion modes were acquired using either an Orbitrap Elite Mass spectrometer or API 3200 Triple Quadrupole System. The Orbitrap Elite Mass spectrometer (Thermofisher Scientific, Bremen, Germany) was equipped with an HESI electrospray ion source (spray voltage 4.0 kV, capillary temperature 275 °C, source heater temperature 80 °C, FTMS resolution 100.000), whereas the API 3200 Triple Quadrupole System (Sciex, Framingham, MA, USA) was equipped with a turbo ion spray source, which performs ionization with an ion spray voltage on 70 eV. During the measurement, the mass/charge range from 50 to 2000 was scanned. 

### 3.2. Plant Material 

The aerial part of *P. obtusifolia* (L.) A. Dietr. was collected in April 2018 at the Leibniz Institute of Plant Biochemistry (IPB), Halle (Saale), Germany from an ornamental plant grown for 20 years on an east oriented window sill. A voucher specimen (ISW010) was deposited in the IPB collection.

### 3.3. Extraction and Isolation

The fresh leaves (267.3 g) of *P. obtusifolia* were dried using a freeze drier and ground to powder form (29.7 g). The powdered material was extracted five times (1.0 L/each) with 80% aqueous methanol at room temperature, yielding 6.4 g of residue after evaporating the solvent in vacuo. The residue was suspended in H_2_O (250 mL) and then successively partitioned with *n*-hexane and ethyl acetate, resulting in the two respective fractions (1.4 g *n*-hexane, 0.8 g EtOAc), as well as an aqueous fraction (3.6 g) (See Figure S20 for the isolation scheme). 

Part of the EtOAc fraction (0.74 g) was chromatographed on a silica gel column and eluted with a stepwise gradient of CH_2_Cl_2_: MeOH (1:0 to 0:1) to give seven fractions (E1-E7). Fraction E1 (32.5 mg) underwent column chromatographic separation over a Sephadex LH-20 column (CH_2_Cl_2_/MeOH 1:1), affording four subfractions (A-D). Further separation of subfraction C (4.3 mg) over silica gel (CH_2_Cl_2_:MeOH 95:5–90:10) yielded becatamide (**4**, 1.4 mg, R*_f_* = 0.25 in 5% MeOH in CH_2_Cl_2_) [21]. Fraction E2 (190.5 mg) was chromatographed over a Sephadex-LH20 column (100% MeOH) to afford an amorphous powder that was identified as *N*-[2-(3,4-dihydroxyphenyl)ethyl]-3,4-dihydroxybenz amide (**3**, 142.5 mg, R*_f_* = 0.37 in 10% MeOH in CH_2_Cl_2_) [20]. Fraction E4 (106.3 mg) was purified over semi-preparative HPLC in a gradient system [H_2_O (A), CH_3_CN (B); 0 min: 5% B > 0–17 min: 85.5% B > 17–20 min: 100% B, UV at 330 nm, 9.8 min], affording compound **2** (14.5 mg).

The *n*-hexane fraction (1.4 g) was chromatographed over a silica gel column and eluted with a stepwise gradient of *n*-hexane, ethyl acetate, and methanol to give six fractions (H1-H6). Fraction H1 (197.4 mg) was further chromatographed over a Sephadex-LH 20 column (100% CH_2_Cl_2_) to afford four subfractions (A-D). Purification of subfraction C (29.1 mg) by silica gel CC (*n*-hex:CH_2_Cl_2_ 7:3) yielded peperobtusin A (**5**, 8.1 mg, R*_f_* = 0.76 in 5% CH_3_OH in CH_2_Cl_2_) [10]. Fraction H2 (279.6 mg) was chromatographed over a Sephadex LH-20 column (CH_2_Cl_2_:MeOH 1:1) to give five subfractions (A-E). Subfraction C (14.7 mg) was purified by silica gel CC (*n*-hex: CH_2_Cl_2_ 3:7–0:1) to afford peperomin B (**6**, 3.1 mg, R*_f_* = 0.04 in CH_2_Cl_2_) [22]. 

The aqueous fraction (2.3 g) was fractionated over a Diaion-HP20 column eluted with an increasing solvent ratio of water and methanol to give five fractions (A1–A5). Fraction A3 (404.7 mg) was chromatographed over a Sepahdex-LH20 column (100% MeOH) to afford seven subfractions (A-G). Subfraction B (55.2 mg) was further separated by silica gel CC (CH_2_Cl_2_:MeOH 7:3) to afford arabinothalictoside (**7**, 14.6 mg, R*_f_* = 0.57 in 30% MeOH in CH_2_Cl_2_) [23]. Subfraction D (69.4 mg) was further chromatographed over a silica gel column (CH_2_Cl_2_:MeOH 8:2–0:1), yielding compound **1** (7.4 mg, R*_f_* = 0.88 in 30% MeOH in CH_2_Cl_2_).

Peperomic ester (trimethyl (1*S*,2*R*)-1-(((*E*)-3-(3,4-dihydroxyphenyl)acryloyl)oxy)- propane-1,2,3-tricarboxylate, **1**): brownish liquid; IR (ATR) *υ*_max_: 3402, 1715 cm^−1^. UV λ_max_ (MeOH) (log e) 218 (4.15), 333 nm (4.21): ^1^H-NMR (400 MHz, CD_3_OD): Table 1; ^13^C-NMR (100 MHz, CD_3_OD): Table 1; negative-ion HR-ESI-MS *m/z* 395.0975 [M − H]^−^ (calcd. for C_18_H_19_O_10_: 395.0984).

Peperoside (*N*-[2-(3,4-dihydroxyphenyl)ethyl]-3,4-dihydroxybenzamide 4′-*O*-*β*-d-glucoside, **2**): white amorphous powder, IR (ATR) *υ*_max_: 3250, 2931, 2360, and 1607 cm^−1^. UV λ_max_ (log e) (MeOH) 290 (3.14), 317 nm (3.32). ^1^H-NMR (400 MHz, CD_3_OD): Table 2; ^13^C-NMR (100 MHz, CD_3_OD): Table 2; negative-ion HR-ESI-MS *m*/*z* 450.1379 [M − H]^−^ (calcd. for C_21_H_24_NO_10_: 450.1406).

### 3.4. Biological activities

#### 3.4.1. Anthelmintic Activity

The anthelmintic bioassay was conducted by applying the method developed by Thomsen et al. using the model organism *C. elegans*, which was previously demonstrated to correlate with anthelmintic activity against parasitic trematodes [33]. The *C. elegans* Bristol N2 wild-type strain was obtained from the Caenorhabditis Genetic Center (CGC), Minnesota University, Minneapolis, USA. The nematodes were grown on petri plates of NGM (Nematode Growth Media) using uracil auxotroph *E. coli* strain OP50 as the food source. The solvent DMSO (2%) and the anthelmintic drug ivermectin (10 μg/mL, nearly 100% dead worms after 30 min incubation) were used as negative and positive controls in all assays, respectively. All assays were carried out in triplicate.

#### 3.4.2. Antifungal Activity

The antifungal activity was examined against the phytopathogenic ascomycetes *B. cinerea* Pars and *S. triciti* Desm. and the oomycete *P. infestans* (Mont.) de Bary in 96-well microtiter plate assays according to protocols from the Fungicide Resistance Action Committee (FRAC) with minor modifications as described by Otto et al. [34]. Briefly, the isolated compounds were tested at the highest concentration of 125 μM, while the solvent DMSO was used as a negative control (max. concentration 2.5%). The commercially used fungicides, epoxiconazole and terbinafine (Sigma-Aldrich, Darmstadt, Germany), served as reference compounds. The pathogen growth was evaluated seven days after inoculation by measurement of the optical density (OD) at ʎ 405 nm with a TecanGENios Pro microplate reader (five measurements per well using multiple reads in 3 × 3 square). Each experiment was carried out in triplicate.

#### 3.4.3. Antibacterial Activity

The isolated compounds (1 and 100 μM) were evaluated against the Gram-negative *A. fischerii* (DSM507) and the Gram-positive *B. subtilis* 168 (DSM 10), as described by dos Santos et al. [35]. The tests were conducted in 96-well plates based on the bioluminescence (*A. fisheri*) or absorption (*B. subtilis*) read-out. Chloramphenicol (100 μM) was used as a positive control to induce complete inhibition of bacterial growth. The results (mean ± standard deviation value, *n* = 6) are given in relation to the negative control (bacterial growth, 1% DMSO without test compound) as relative values (percent inhibition). Negative values indicate an increase in bacterial growth. 

#### 3.4.4. Cytotoxic Activity

The cytotoxicity and impact on the metabolic cell viability of isolated compounds at 10 nM and 10 μM was evaluated against PC-3 (human prostate adenocarcinoma) and HT-29 (human colorectal adenocarcinoma) cancer cell lines. Both cell lines were purchased from ATCC (Manassas, VA, USA). The cell culture medium RPMI 1640, the supplements FCS and L-glutamine, as well as PBS and trypsin/EDTA were purchased from Capricorn Scientific GmbH (Ebsdorfergrund, Germany). Culture flasks, multi-well plates, and further cell culture plastics were from Greiner Bio-One GmbH (Frickenhausen, Germany) and TPP (Trasadingen, Switzerland), respectively. Resazurin used for the cell viability assays was purchased from Sigma-Aldrich GmbH (Taufkirchen, Germany). PC-3 and HT-29 cells were cultured in RPMI 1640 media supplemented with 10% heat-inactivated FCS, 2 mM L-glutamine, and 1% penicillin/streptomycin in a humidified atmosphere with 5% CO_2_ at 37 °C. Routinely, cells were cultured in T-75 flasks until reaching subconfluency (~80%); subsequently, cells were harvested by washing with PBS and detaching by using trypsin/EDTA (0.05% in PBS) prior to cell passaging and seeding for subculturing and assays in 96-well plates, respectively [36].

The cell handling and assay techniques were in accordance with the method described by Khan et al. [36]. In brief, antiproliferative and cytotoxic effects of the compounds were investigated by performing colorimetric MTT (3-(4,5-dimethylthiazol-2-yl)-2,5-diphenyltetrazolium bromide) and CV (crystal violet)-based cell viability assays (Sigma-Aldrich, Taufkirchen, Germany), respectively. For this purpose, cells were seeded in low densities in 96-well plates using the aforementioned cell culture medium. The cells were allowed to adhere for 24 h, followed by the 48 h compound treatment. Based on 20 mM DMSO stock solutions, the compounds were serially diluted in standard growth media to reach final concentrations of 100, 50, 25, 12.5, 6.25, 3.125, and 1.56 µM for cell treatment. For control measures, cells were treated in parallel with 125 µM digitonin (positive control, for data normalization set to 0% cell viability). Each data point was determined in technical quadruplicates and two independent biological replicates. As soon as the 48 h incubation was finished, cell viability was measured.

For the MTT assay, cells were washed once with PBS, followed by incubation with MTT working solution (0.5 mg/mL MTT in culture medium) for 1 h under standard growth conditions. After discarding the MTT solution, DMSO was added in order to dissolve the formed formazan, followed by measuring formazan absorbance at 570 nm, and additionally, at the reference/background wavelength of 670 nm by using a SpectraMax M5 multi-well plate reader (Molecular Devices, San Jose, CA, USA).

For the CV assay, cells were washed once with PBS and fixed with 4% paraformaldehyde (PFA) for 20 min at room temperature (RT). After discarding the PFA solution, the cells were left to dry for 10 min and then stained with 1% crystal violet solution for 15 min at RT. The cells were washed with water and dried overnight at RT. Afterwards, acetic acid (33% in ultrapure water) was added to the stained cells and absorbance was measured at 570 nm and 670 nm (reference wavelength) using a SpectraMax M5 multi-well plate reader (Molecular Devices, San Jose, CA, USA). For data analyses, GraphPad Prism version 8.0.2, SigmaPlot 14.0 and Microsoft Excel 2013 were used.

The results are shown as a percentage of the control values obtained from untreated cultures, i.e. cell viability in percent.

## 4. Conclusions

The present investigation of a methanol extract from the leaves of *P. obtusifolia* led to the isolation of two new compounds named peperomic ester (**1**) and peperoside (**2**), along with five known compounds. The isolated compounds were evaluated for their anthelmintic, antifungal, antibacterial, and anticancer activities. Peperoside (**2**) and its related compounds (**3, 4**) have the advantage of low activity against all organisms/cells tested. Since similar phenethylamides show taste and neuroactive properties [37,38]—which were not part of this study—their use in such tests, which usually include human panels or animals, appears acceptable.

The known peperobtusin A (**5**) was the most active compound against *B. subtilis* and showed an antiproliferative and cytotoxic effect in the lower micromolar concentration range towards human PC-3 (prostate) and HT-29 (colon) cancer cell lines. The observed antibacterial activity supports the traditional use of *P. obtusifolia* as a skin cleanser. These findings suggest that *P. obtusifolia* might have some general toxicity and thus should be used with care in applications where cytotoxicity is not desired. However, prenylated benzopyran derivatives structurally related to **5** were shown to exhibit selective activity against breast cancer cells but did not influence normal breast cells [39]. The impact of this compound class on the mitochondrial respiratory chain [39,40] and their function as PPAR agonists may play a role in compound selectivity [41]. Thus, more extensive and detailed studies on the anticancer activity of natural prenylated benzopyrans should be undertaken in order to provide an understanding of structural requirements (SAR) for such cytotoxic (or other) activity. 

## Figures and Tables

**Table 1:** ^1^H- and ^13^C-NMR data of **1** (in CD_3_OD; δ in ppm, *J* in Hz).

No.	δ (H) ^a^	δ (C) ^b^	HMBC
1	-	127.4	
2	7.06 (d, *J* = 2.1)	115.2	
3	-	146.8	
4	-	150.0	
5	6.79 (d, *J* = 8.2)	116.5	
6	6.97 (dd, *J* = 8.2, 2.1)	123.3	
7	7.59 (d, *J* = 15.8)	148.6	C1, C2, C6, C8, C9
8 9	6.29 (d, *J* = 15.8) -	113.4 167.5	C1, C7, C9
1’	5.46 (d, *J* = 3.9)	72.6	C2’, C3’, C5’, C6’, C9’
2’	3.62 (ddd, *J* = 9.0, 5.5, 3.9)	44.2	C1’, C3’, C4’, C5’, C6’
3’a	2.85 (d, *J* = 17.2, 9.0)	32.8	C2’, C1’, C4’, C6’
3’b	2.66 (d, *J* = 17.2, 5.5)	32.8	C2’, C1’, C4’, C6’
4’	-	173.2	
5’	-	170.2	
6’	-	172.1	
OMe	3.73	52.9	C6’
OMe OMe	3.77 3.68	53.1 53.4	C5’ C4’

^a^ Recorded at 400 MHz. ^b^ Recorded at 100 MHz.

**Table 2:** ^1^H- and ^13^C-NMR data of **2** (in CD_3_OD; δ in ppm, *J* in Hz).

No.	δ (H) ^a^	δ (C) ^b^	HMBC
1	-	132.2	
2	6.70 (d, *J* = 2.1)	117.0	C1, C3, C4, C6, C7
3	-	144.7	
4	-	146.2	
5	6.69 (d, *J* = 8.0)	116.5	C1, C3, C4, C6, C7
6	6.59 (dd, *J* = 8.0, 2.1)	121.1	C2, C3, C7
7	2.75 (t, *J* = 7.6)	35.9	C1, C2, C6, C8
8a	3.57 (dt, *J* = 13.2, 7.6)	42.8	C1, C7, C7’
8b	3.50 (dt, *J* = 13.2, 7.6)	42.8	C1, C7, C7’
1’	-	126.3	
2’	7.25 (d, *J* = 3.1)	120.7	C1’, C3’, C4’, C5’, C6’, C7’
3’	-	154,3	
4’	-	150.1	
5’	7.24 (d, *J* = 8.8)	117.3	C1’, C3’, C4’, C6’
6’	6.86 (dd, *J* = 8.8, 3.1)	120.3	C1’, C3’, C4’, C5’
7’	-	167.8	
1’’	4.79 (d, *J* = 7.5)	105.1	C4’, C3’’, C5’’
2’’	3.38 (m)	74.9	
3’’	3.41 (m)	78.1	
4’’	3.38 (m)	78.5	
5’’	3.39 (m)	71.3	
6a’’	3.70 (dd, *J* = 12.0, 5.0)	62.5	
6b’’	3.89 (d, *J* = 12.0)	62.5	

^a^ Recorded at 400 MHz. ^b^ Recorded at 100 MHz.

**Table 3:** Anthelmintic (*Caenorhabditis elegans*), antifungal (*Botrytis cinerea*, *Septoria tritici* and *Phytophthora infestans*), and antibacterial (*Bacillus subtilis*, *Aliivibrio fisheri*) activities of isolated compounds (**1**–**7**) from *P. obtusifolia*.

	Anthelmintic Assay	Antifungal Assays			Antibacterial Assays	
	Mortality [%]	Growth Inhibition [%] ^a^			Growth Inhibition [%] ^a^	
	*C. elegans*	*B. cinerea*	*S. tritici*	*P. infestans*	*B. subtilis*	*A. fischeri*
**Compound**	200 µM	125 µM	125 µM	125 µM	100 µM ^b^	100 µM ^b^
**1**	1.9 ± 0.8	−13.6 ± 17.3	−12.5 ± 17.9	10.1 ± 2.3	8.0 ± 19.0	−5.6 ± 2.60
**2**	0.0 ± 0.0	−27.6 ± 7.6	21.8 ± 8.9	−51.5 ± 16.0	7.0 ± 15.0	−3.1 ± 1.80
**3**	0.0 ± 0.0	−18.5 ± 3.1	−40.0 ± 35.2	56.2 ± 10.3	7.0 ± 2.8	−211.6 ± 27.6
**4**	2.9 ± 1.4	−32.3 ± 28.7	-9.8 ± 8.3	−66.4 ± 30.4	−21.0 ± 28.0	−35.9 ± 19.4
**5**	4.8 ± 1.4	27.0 ± 25.8	46.9 ± 8.3	−6.0 ± 64.4	90.0 ± 2.0	−32.7 ± 12.7
**6**	2.0 ± 1.7	51.4 ± 22.3	−12.9 ± 2.3	−9.4 ± 27.4	−5.0 ± 51.0	30.4 ± 8.4
**7**	1.7 ± 2.4	2.8 ± 19.5	−7.2 ± 1.9	17.1 ± 12.1	5.0 ± 17.0	1.6 ± 3.0
Pos. control	10 μg/mL ivermectin	125 µM epoxiconazole		125 µM terbinafine	100 µM chloramphenicol	
	98.7 ± 1.9	92.0 ± 1.4		96.8 ± 1.2	99.0 ± 0	100.0 ± 0

^a^ Negative values indicate an increase in fungal or bacterial growth in comparison to the negative control (0% inhibition); ^b^ Growth inhibition rates below 50% indicate IC_50_ values > 100 µM.

**Table 4:** Antiproliferative and cytotoxic activities of isolated compounds (**1**–**7**) from *P. obtusifolia* against human prostate (PC-3) and colorectal (HT-29) cancer cell lines.

	Metabolic cell Viability (MTT)/Cytotoxicity (CV) Assays			
	Cell Viability/Survivals [%]			
	PC-3 (MTT Assay)	PC-3 (CV Assay)	HT-29 (MTT Assay)	HT-29 (CV Assay)
**Compound**	10 µM	10 µM	10 µM	10 µM
**1**	101.4 ± 5.8	97.5 ± 4.1	100.3 ± 4.4	91.0 ± 5.1
**2**	84.1 ± 4.9	84.0 ± 6.9	74.9 ± 6.5	78.2 ± 4.4
**3**	90.6 ± 3.3	98.3 ± 1.3	82.6 ± 4.8	100.3 ± 3.7
**4**	114.4 ± 1.2	92.9 ± 5.0	111.9 ± 1.8	89.0 ± 3.0
**5**	2.4 ± 6.3	−3.4 ± 24.7	59.7 ± 20.5	82.5 ± 4.3
**6**	120.9 ± 4.3	93.7 ± 2.6	117.1 ± 3.0	88.2 ± 3.9
**7**	113.7 ± 4.0	112.2 ± 4.7	115.3 ± 3.9	107.5 ± 2.9

Digitonin (125 µM) was used as a positive control, compromising the cells to the point of 0% cell viability/cancer cell survival after 48 h.

## Data Availability

The data presented in this study are available on request from the corresponding author.

## Supplementary Materials

The following supporting information can be downloaded at: https://www.mdpi.com/article/10.3390/molecules27144363/s1, Figure S1: ^1^H NMR spectrum of compound **1** (400 MHz, MeOH-*d*_4_), Figure S2: ^13^C NMR spectrum of compound **1** (100 MHz, MeOH-*d*_4_), Figure S3: HSQC spectrum of compound **1** (400 MHz, MeOH-*d*_4_), Figure S4: HMBC spectrum of compound **1** (400 MHz, MeOH-*d*_4_), Figure S5: ^1^H-^1^H COSY spectrum of compound **1** (400 MHz, MeOH-*d*_4_), Figure S6: UV spectrum of compound **1** in MeOH, Figure S7: IR spectrum of compound **1** in MeOH, Figure S8: ESI-HRMS spectrum of compound **1** in negative ion mode, Figure S9: HRMS spectrum from ethanol extract of *P. obtusifolia* with selected molecular ion of *m/z* 395.0979 [M − H]^−^, Figure S10: ^1^H NMR spectrum of compound **2** (400 MHz, MeOH-*d*_4_), Figure S11: ^13^C NMR spectrum of compound **2** (100 MHz, MeOH*-d*_4_), Figure S12: DEPT135 spectrum of compound **2** (100 MHz, MeOH*-d*_4_), Figure S13: HSQC spectrum of compound **2** (400 MHz, MeOH-*d*_4_), Figure S14: HMBC spectrum of compound **2** (400 MHz, MeOH-*d*_4_), Figure S15: ^1^H-^1^H COSY spectrum of compound **2** (400 MHz, MeOH-*d*_4_), Figure S16: 1D-ROESY spectrum of compound **2** (400 MHz, MeOH*-d*_4_), Figure S17: UV spectrum of compound **2** in MeOH, Figure S18: IR spectrum of compound **2** in MeOH, Figure S19: ESI-HRMS spectrum of compound **2** in negative ion mode, Figure S20. Isolation scheme for compounds **1**–**7**, Table S1: Polarimeter data of compound **1**.
