# Supplementary material for: Bioactive Phenolic Compounds from Peperomia obtusifolia
Source: Molecules. 2022 Jul 7;27(14):4363. doi: 10.3390/molecules27144363 (PMC9315869; doi:10.3390/molecules27144363)
Supplement: Supplementary file 1 [file molecules-27-04363-s001.zip › molecules-1785979-supplementary.pdf]

Supporting Information

Bioactive phenolic compounds from *Peperomia obtusifolia*

Ismail Ware[1,2], Katrin Franke[1*], Hidayat Hussain[1], Ibrahim Morgan[1], Robert Rennert[1], Ludger A. Wessjohann[1*]

[1] Department of Bioorganic Chemistry, Leibniz Institute of Plant Biochemistry, *D*-06120 Halle (Saale), Germany; IsmailBin.Ware@ipb-halle.de (I.W.); Katrin.Franke@ipb-halle.de (K.F.); Hidayat.Hussain@ipb-halle.de (H.H.); Ibrahim.Morgan@ipb-halle.de (I.M.); Robert.Rennert@ipb-halle.de (R.R.); Ludger.Wessjohann@ipb-halle.de (L.A.W.)
[2] Institute of Bioproduct Development, Universiti Teknologi Malaysia, 81310 UTM, Johor Bahru, Johor, Malaysia.

* Correspondence: Katrin.Franke@ipb-halle.de (K.F.); wessjohann@ipb-halle.de (L.A.W.) Tel.: +49-345-5582-1380 (K.F.); +49-345-5582-1300 (L.A.W.)

TABLE OF CONTENTS **PAGE**

Figure S1: ^{1}H NMR spectrum of compound **1** (400 MHz, MeOH-d_4)....2
Figure S2: ^{13}C NMR spectrum of compound **1** (100 MHz, MeOH-d_4)....2
Figure S3: HSQC spectrum of compound **1** (400 MHz, MeOH-d_4). 3
Figure S4: HMBC spectrum of compound **1** (400 MHz, MeOH-d_4). 3
Figure S5: ^{1}H-^{1}H COSY spectrum of compound **1** (400 MHz, MeOH-d_4)....4
Figure S6: UV spectrum of compound **1** in MeOH. 5
Figure S7: IR spectrum of compound **1** in MeOH. 5
Figure S8: ESI-HRMS spectrum of compound **1** in negative ion mode. 6
Figure S9: HRMS spectrum from ethanol extract of *P. obtusifolia* with selected molecular ion of *m/z* 395.0979 $[M-H]^-$....6
Table S1: Polarimeter data of compound **1**.... 7
Figure S10: ^{1}H NMR spectrum of compound **2** (400 MHz, MeOH-d_4)....8
Figure S11: ^{13}C NMR spectrum of compound **2** (100 MHz, MeOH-d_4)....8
Figure S12: DEPT135 spectrum of compound **2** (100 MHz, MeOH-d_4). 9
Figure S13: HSQC spectrum of compound **2** (400 MHz, MeOH-d_4). 9
Figure S14: HMBC spectrum of compound **2** (400 MHz, MeOH-d_4). 10
Figure S15: ^{1}H-^{1}H COSY spectrum of compound **2** (400 MHz, MeOH-d_4)....10
Figure S16: 1D-ROESY spectrum of compound **2** (400 MHz, MeOH-d_4). 11
Figure S17: UV spectrum of compound **2** in MeOH. 11
Figure S18: IR spectrum of compound **2** in MeOH. 12
Figure S19: ESI-HRMS spectrum of compound **2** in negative ion mode. 12
Figure S20: Isolation scheme for compounds **1**-**7**....12

Trimethyl (1*S*,2*R*)-1-(((*E*)-3-(3,4-dihydroxy phenyl)acryloyl)oxy)propane-1,2,3-tricarboxylate (peperomic ester, **1**)

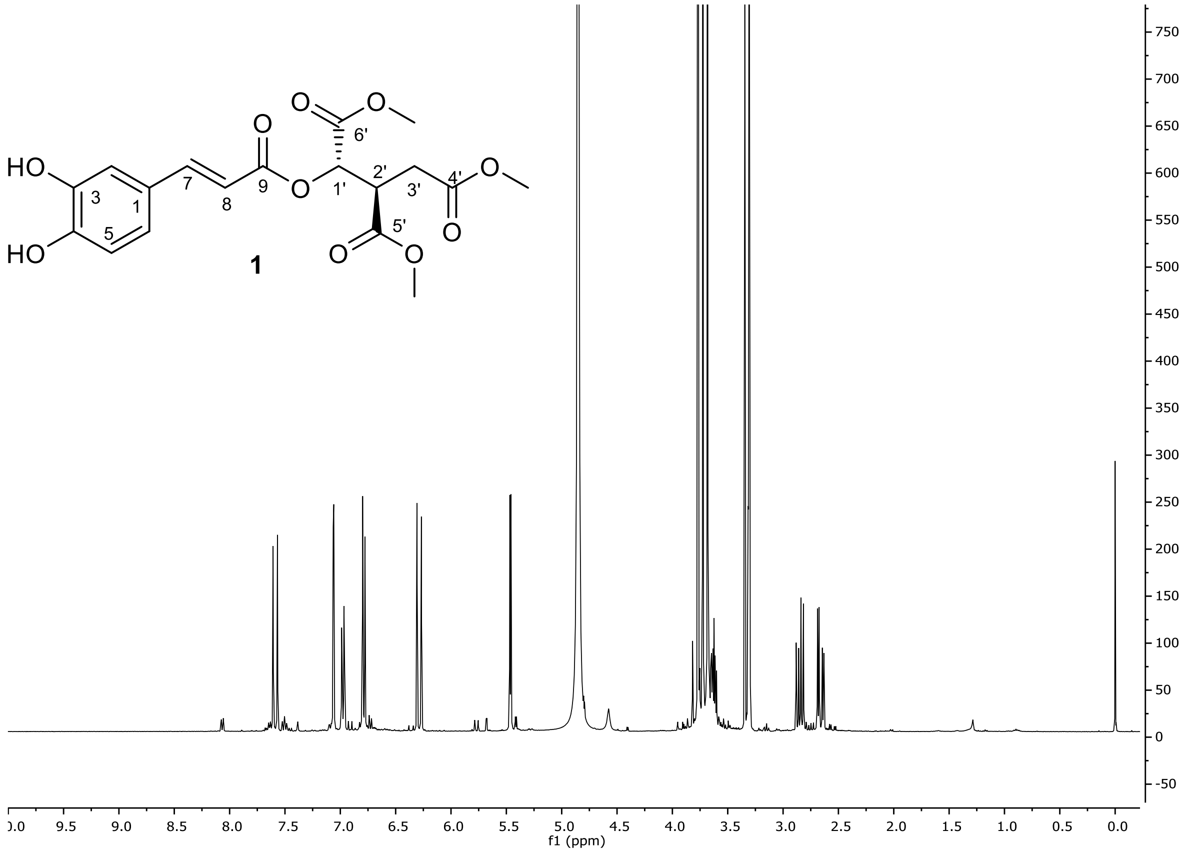

Figure S1: ^{1}H NMR spectrum of compound **1** (400 MHz, MeOH-d_4).

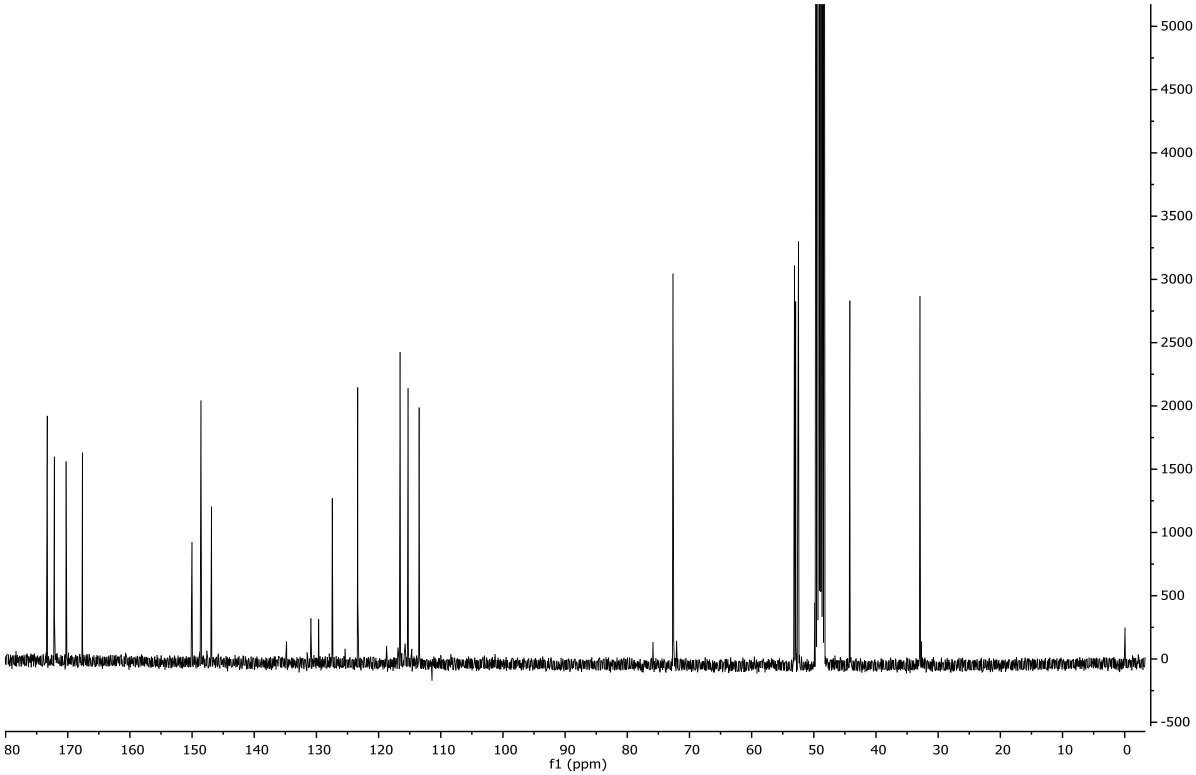

Figure S2: ^{13}C NMR spectrum of compound **1** (100 MHz, MeOH-d_4).

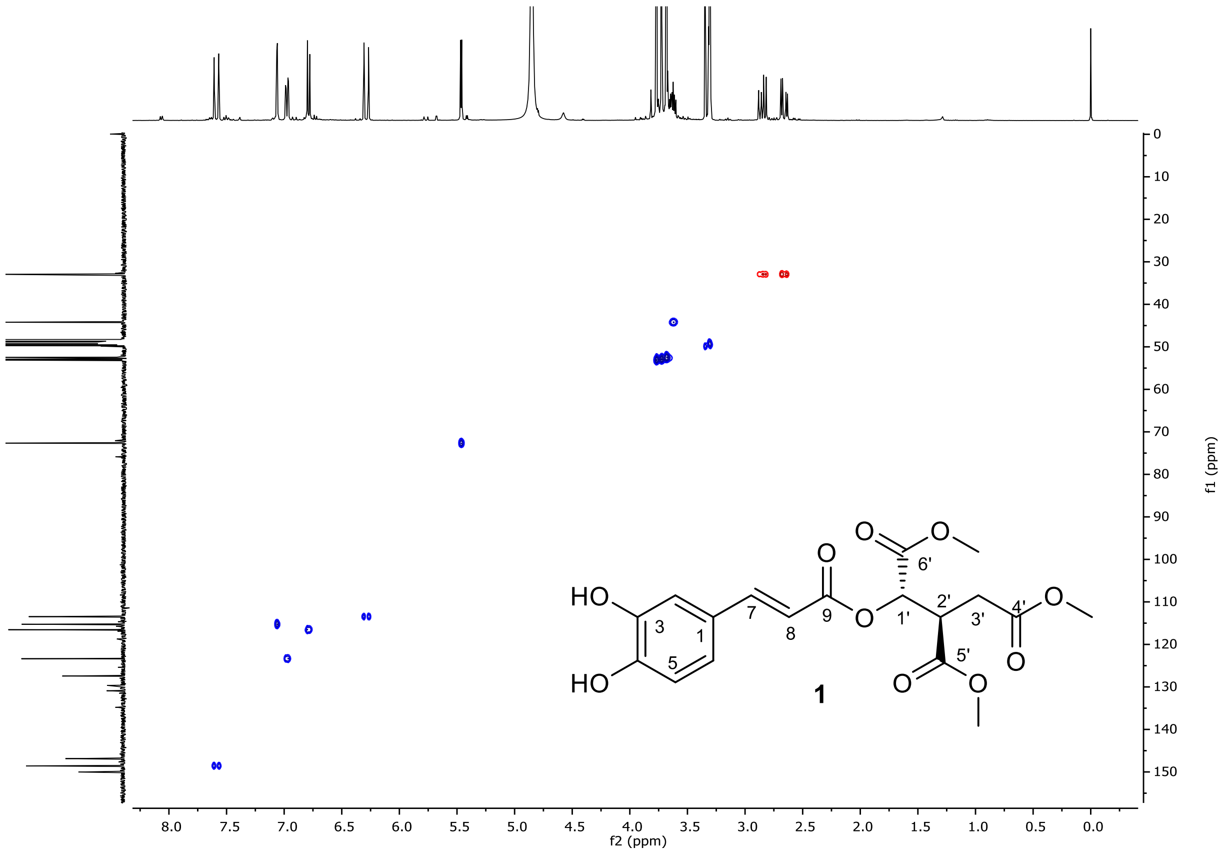

Figure S3: HSQC spectrum of compound **1** (400 MHz, MeOH-*d*4).

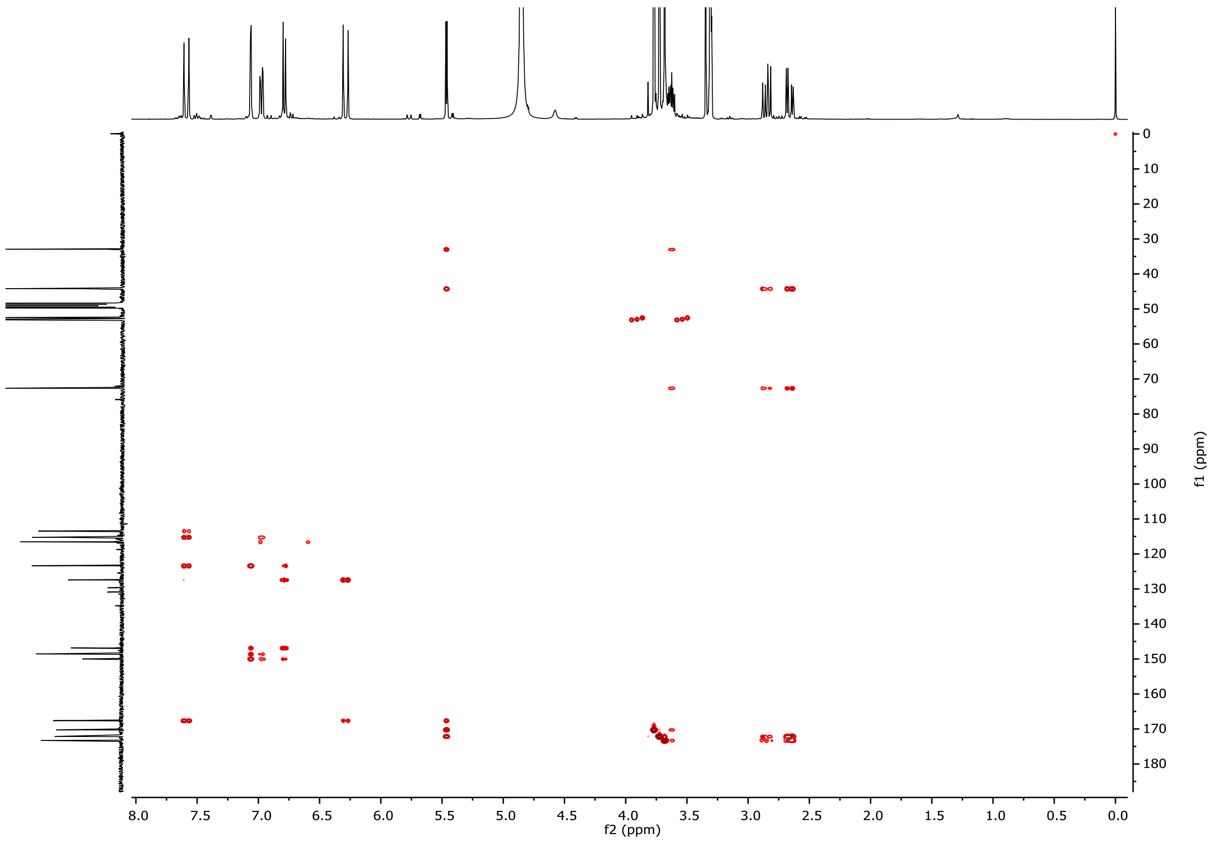

Figure S4: HMBC spectrum of compound **1** (400 MHz, MeOH-*d*4).

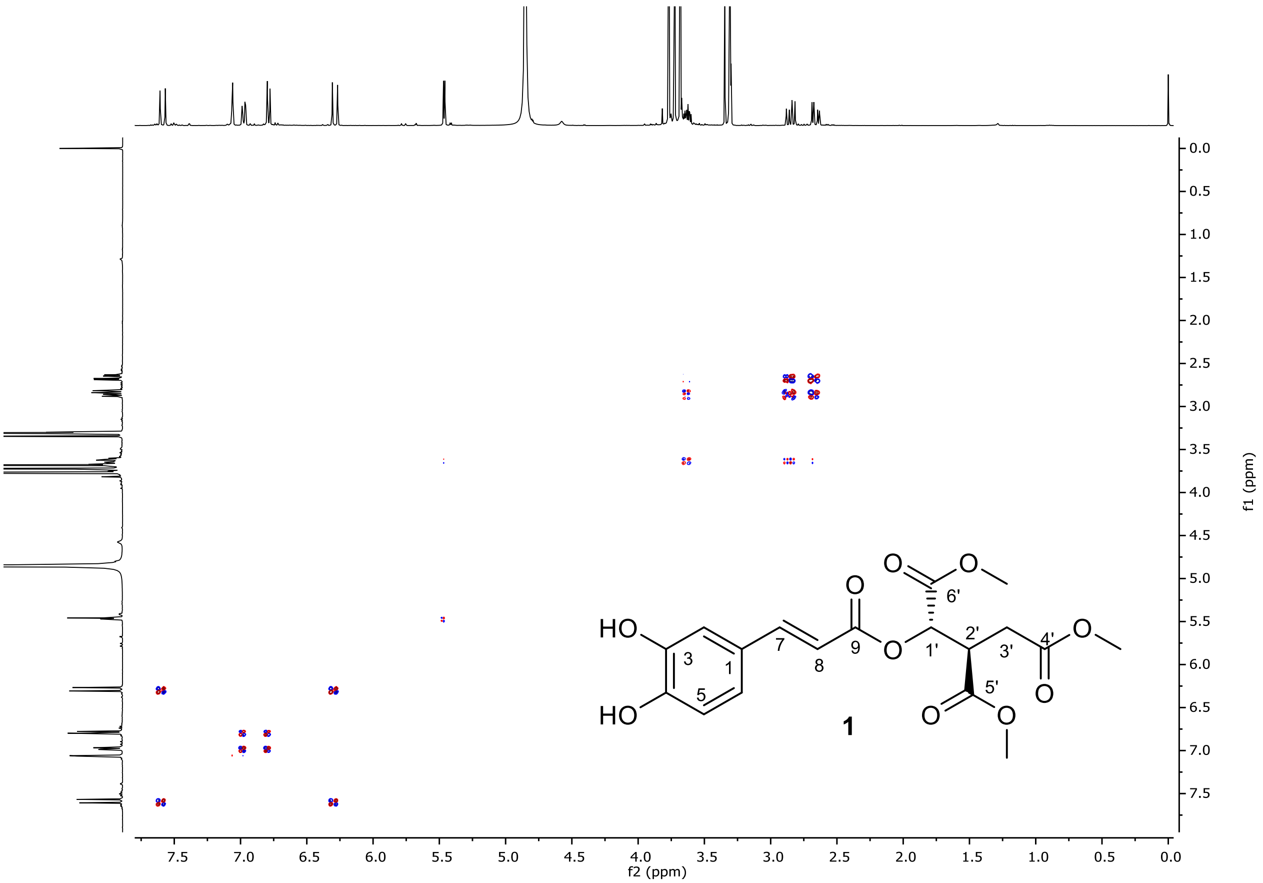

Figure S5: ^1H-^1H COSY spectrum of compound **1** (400 MHz, MeOH-*d*4).

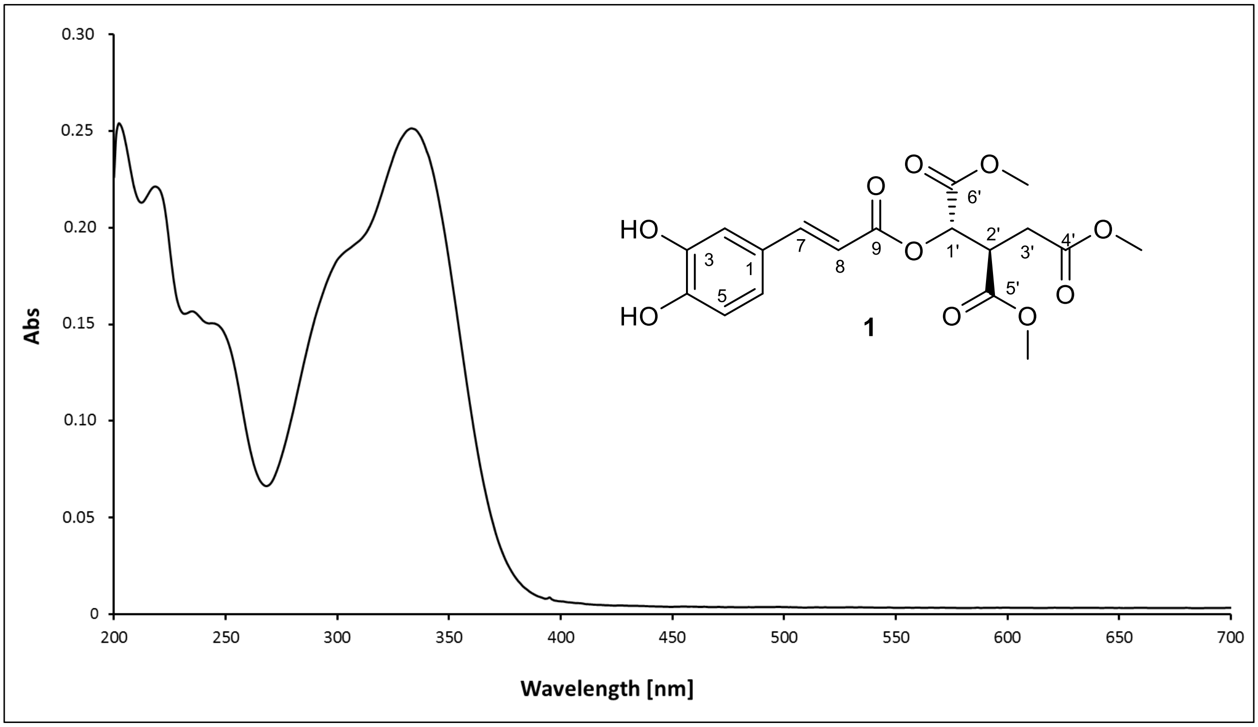

Figure S6: UV spectrum of compound **1** in MeOH.

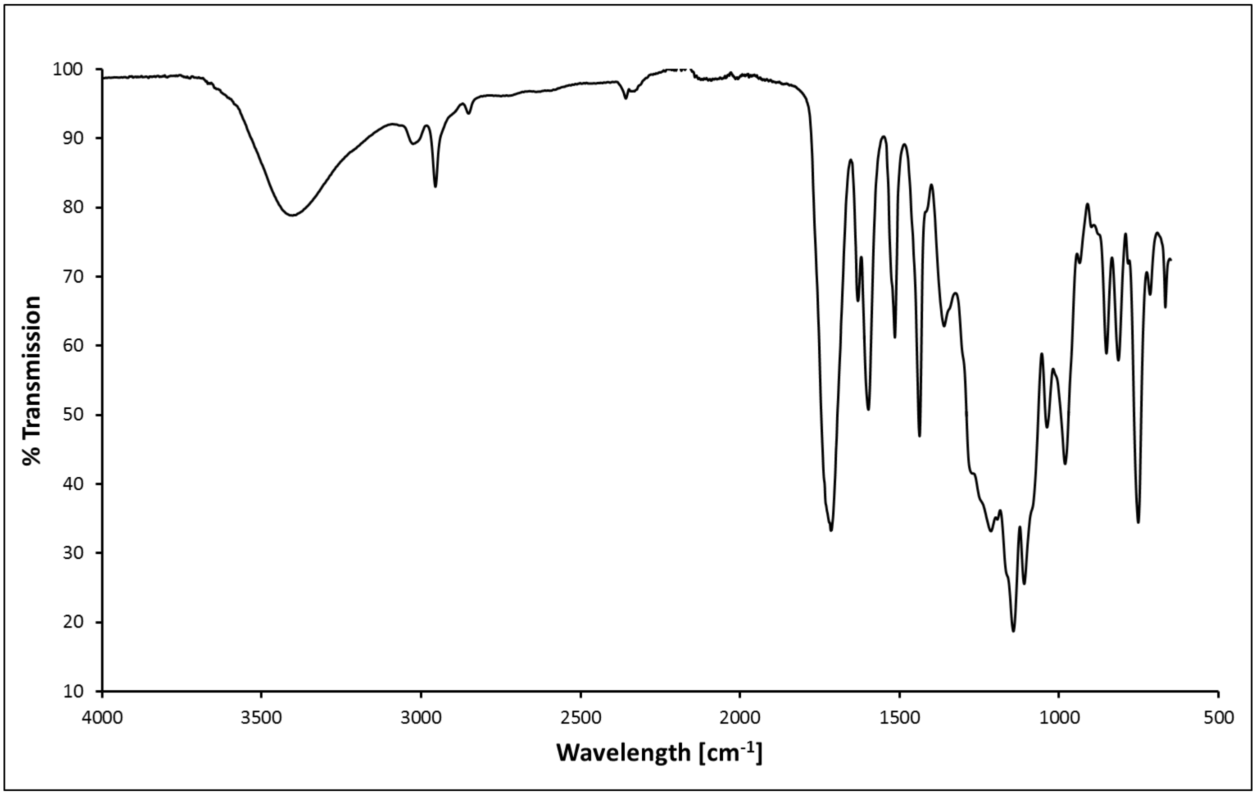

Figure S7: IR spectrum of compound **1** in MeOH.

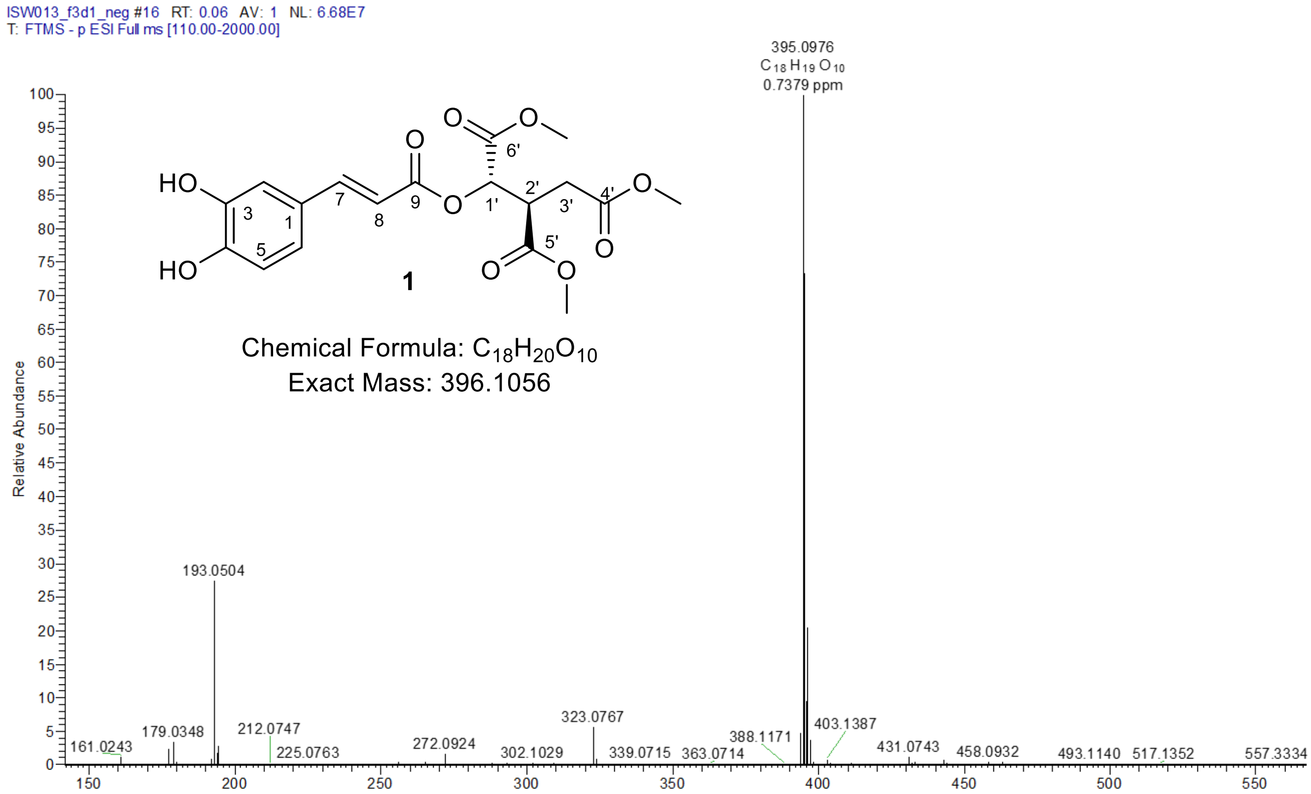

Figure S8: ESI-HRMS spectrum of compound **1** in negative ion mode.

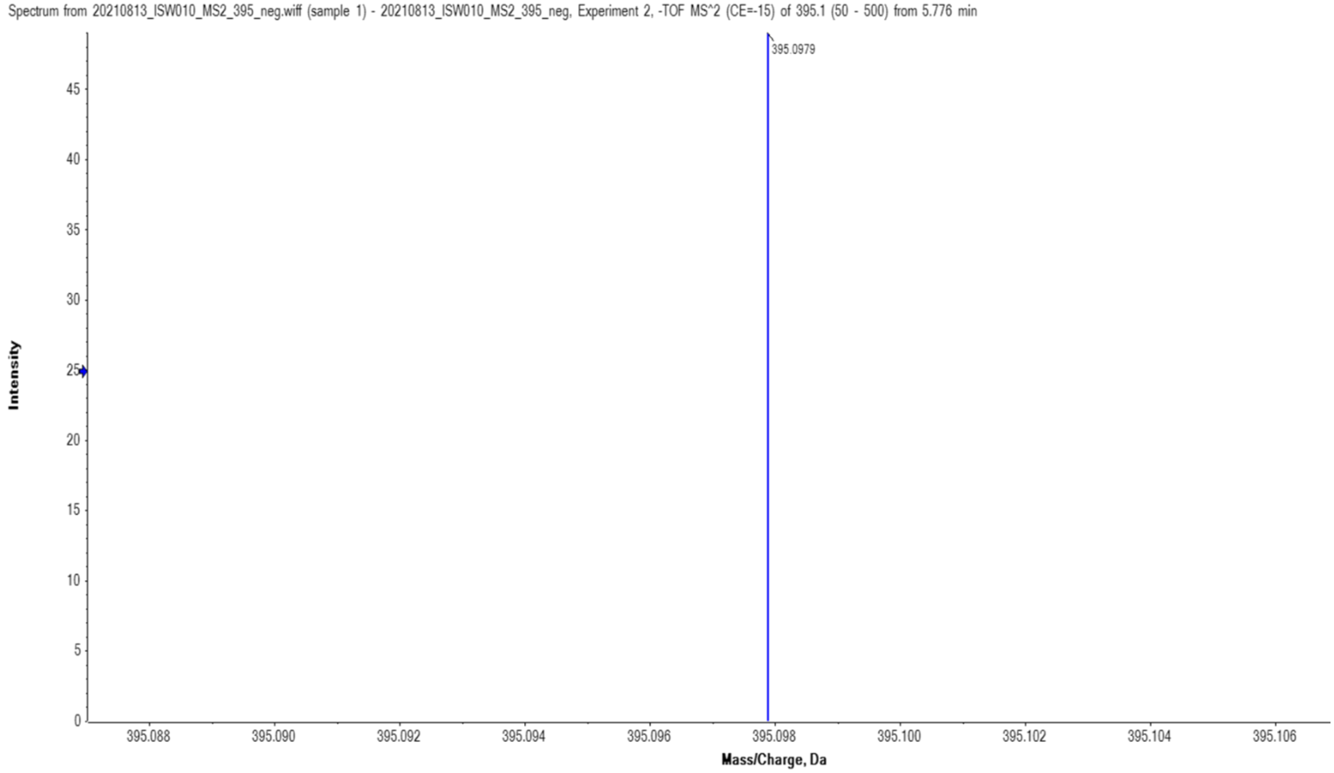

Figure S9: HRMS spectrum from ethanol extract of *P. obtusifolia* with selected molecular ion of *m/z* 395.0979 [M-H]- (**1**).

Table S1: Polarimeter data of compound **1**.

Light Source	WI
Wavelength	589 nm
Sample Aperture	Ø 3.0
Light Aperture	Ø 1.0
D.I.T.	5 sec
Cycle Times	20
Cycle Interval	5 sec
Path Length	100 mm
Concentration	0.1545 W/V%
Factor	1.0000
Temp. Correct	0
**Sample**	**ISW013_f3d1**
Comment	3.09 mg / 2 ml Methanol
**Optical Rotation**	
Average	-0.0180 deg
S.D.	0.0004 deg
R.S.D.	2.2603 %
**Specific O.R.**	
Average	-11.6634
S.D.	0.2636
Temperatur	21.9° C

N-[2-(3,4-dihydroxyphenyl)ethyl]-3,4-dihydroxybenzamide 4′-*O*-*β*-D-glucoside (peperoside, **2**)

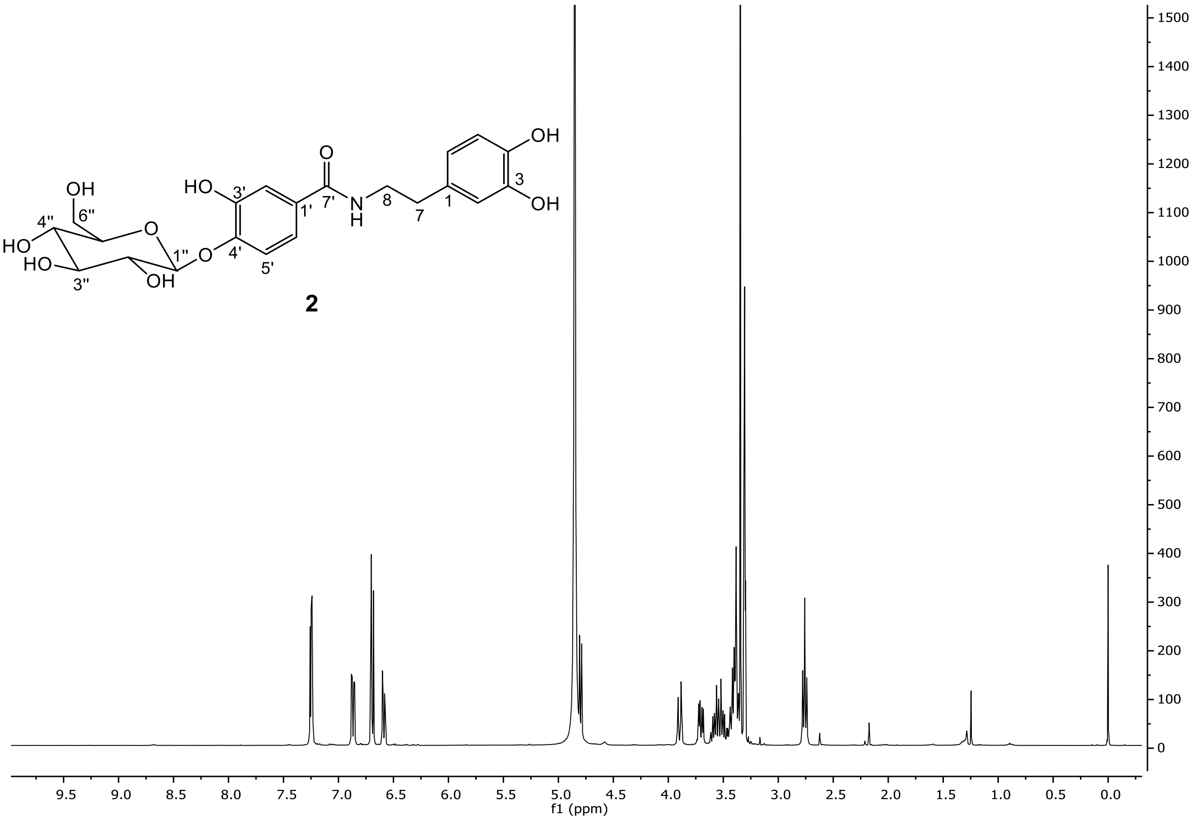

Figure S10: ^{1}H NMR spectrum of compound **2** (400 MHz, MeOH-d_4).

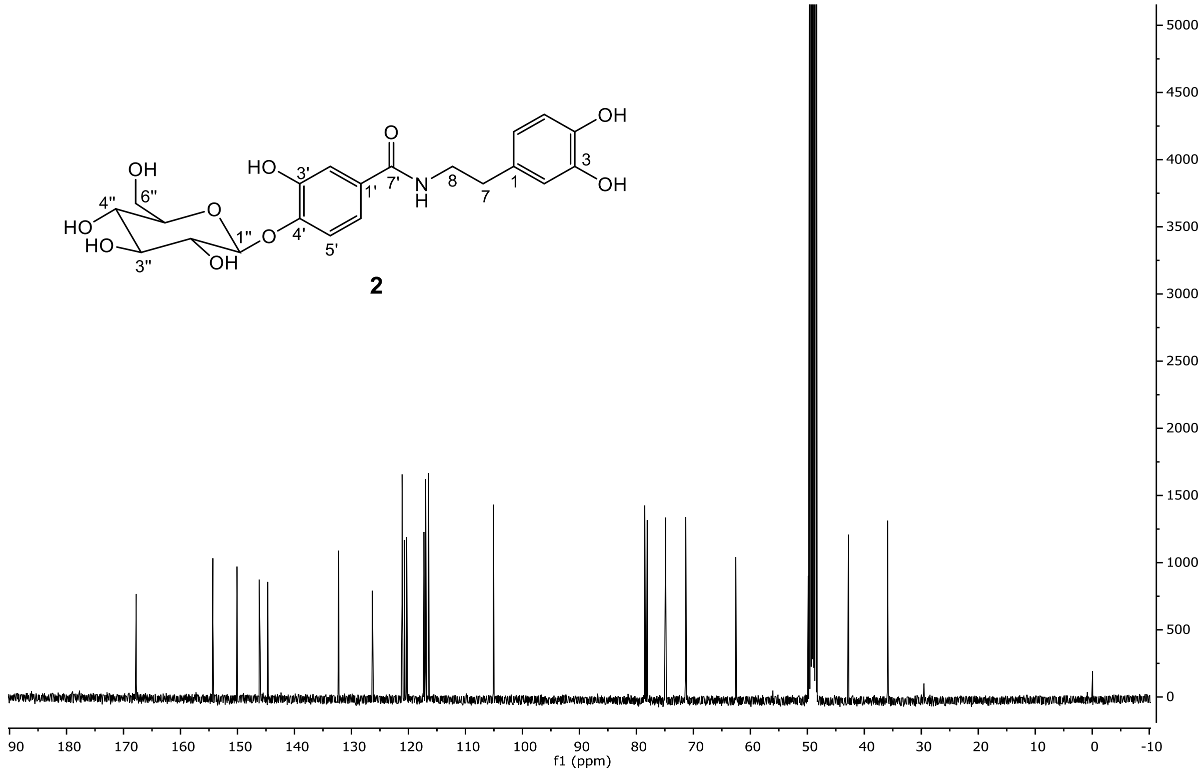

Figure S11: ^{13}C NMR spectrum of compound **2** (100 MHz, MeOH-d_4).

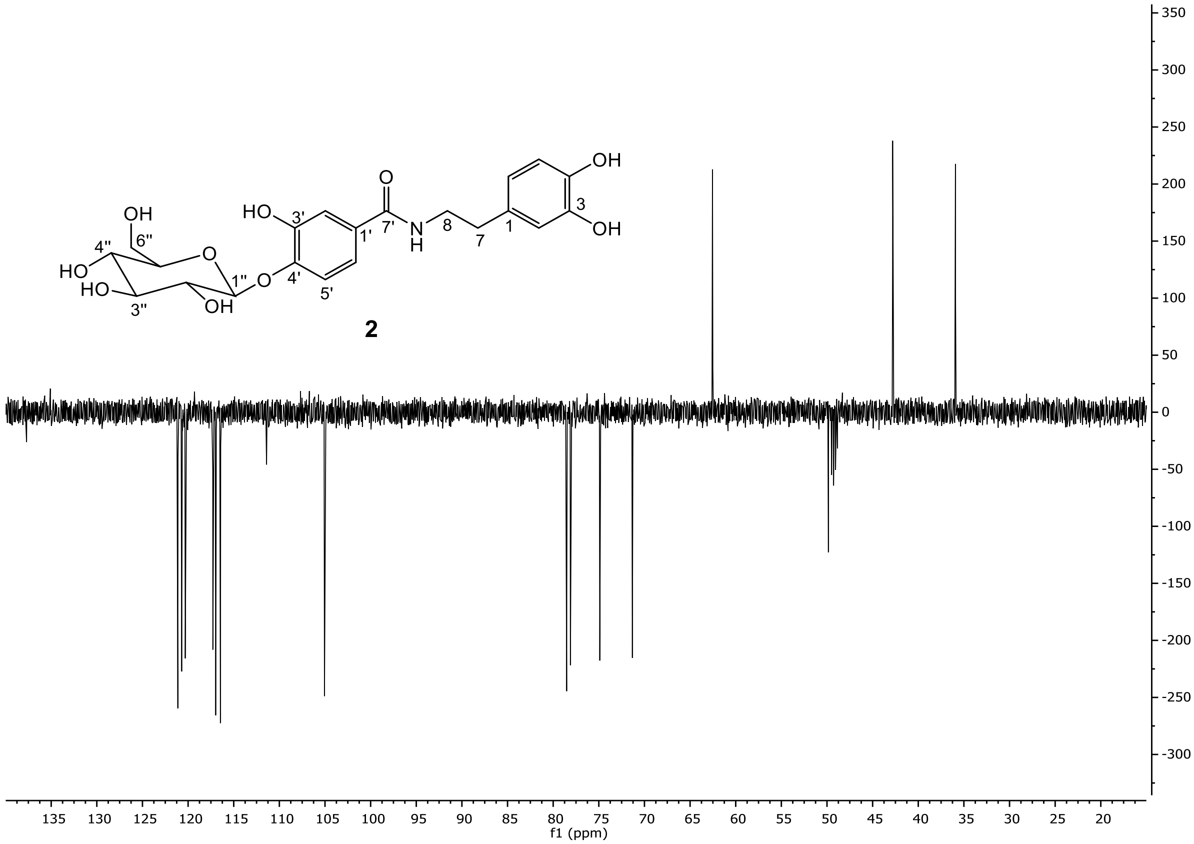

Figure S12: DEPT135 spectrum of compound **2** (100 MHz, MeOH-d_4).

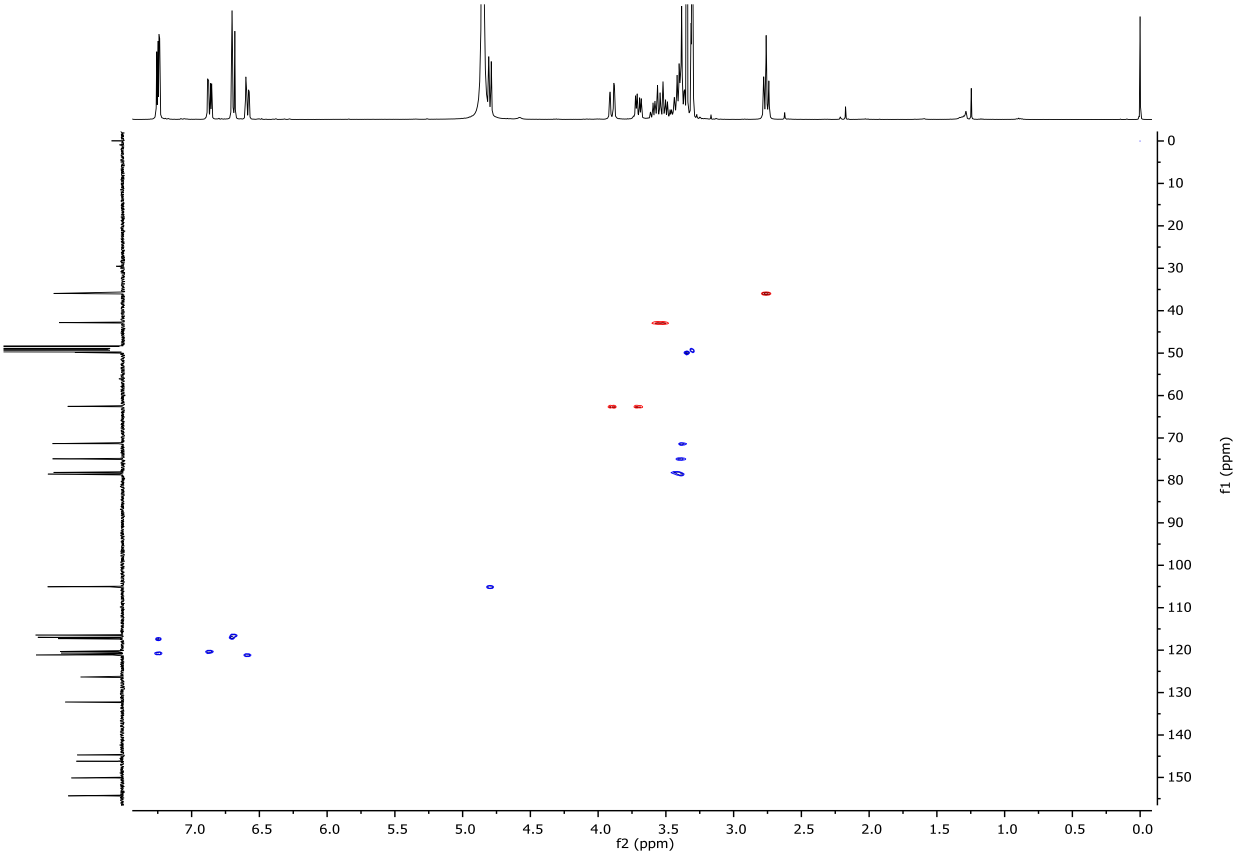

Figure S13: HSQC spectrum of compound **2** (400 MHz, MeOH-d_4).

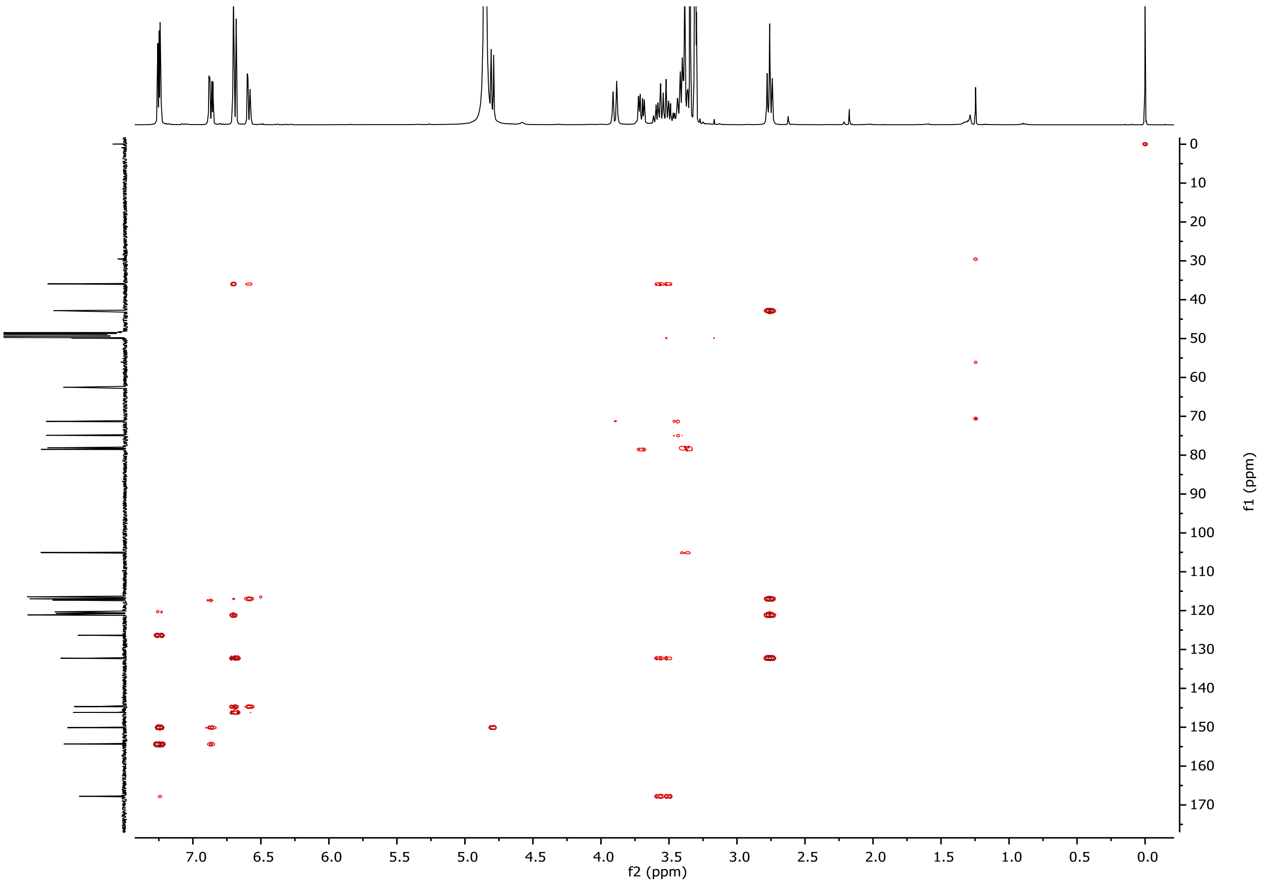

Figure S14: HMBC spectrum of compound **2** (400 MHz, MeOH-d_4).

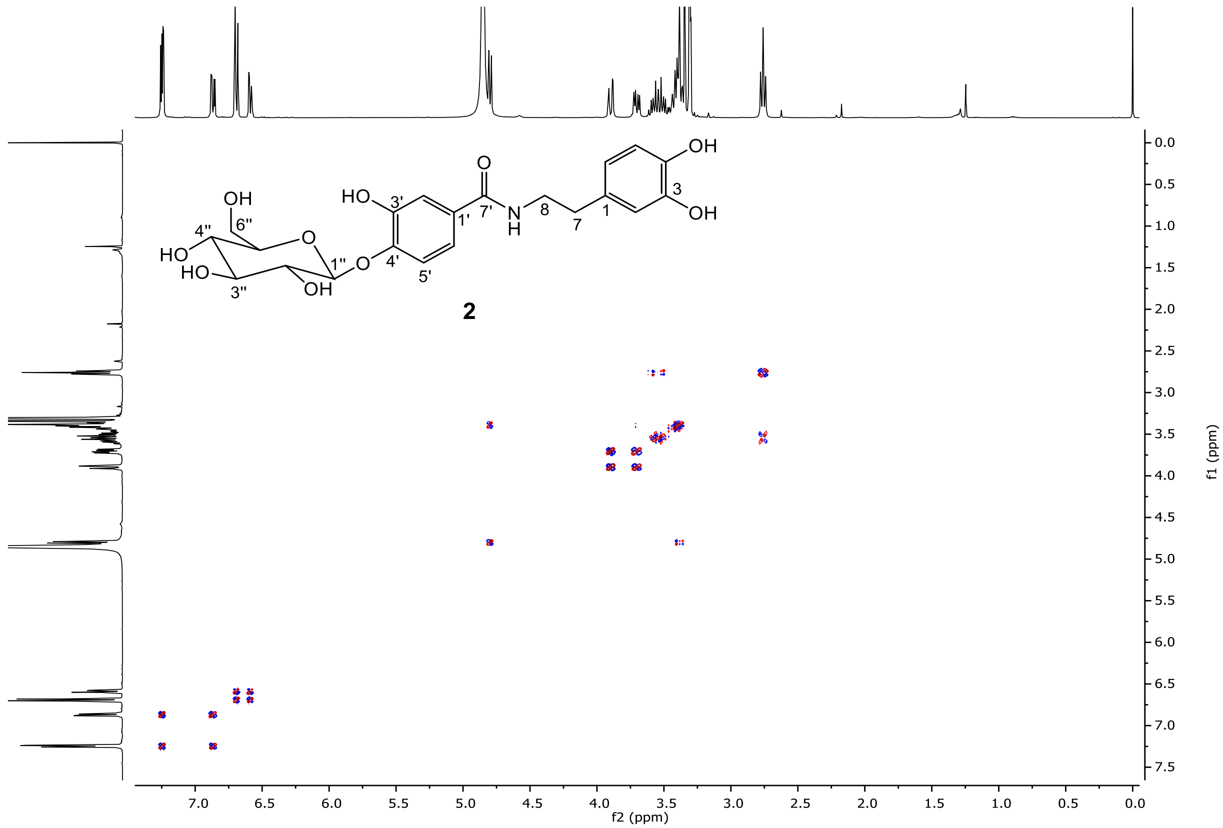

Figure S15: ^{1}H-^{1}H COSY spectrum of compound **2** (400 MHz, MeOH-d_4).

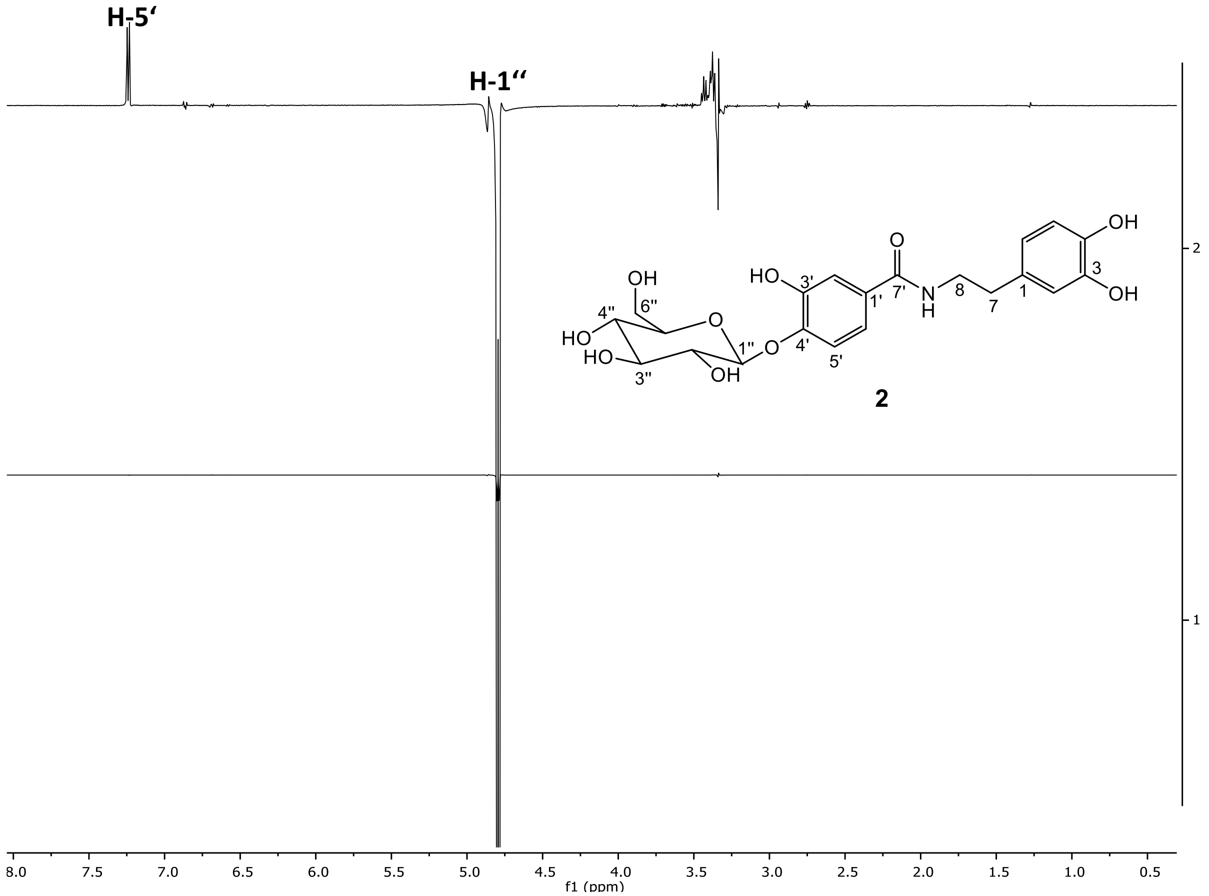

Figure S16: 1D-ROESY spectrum of compound **2** (400 MHz, MeOH-d_4).

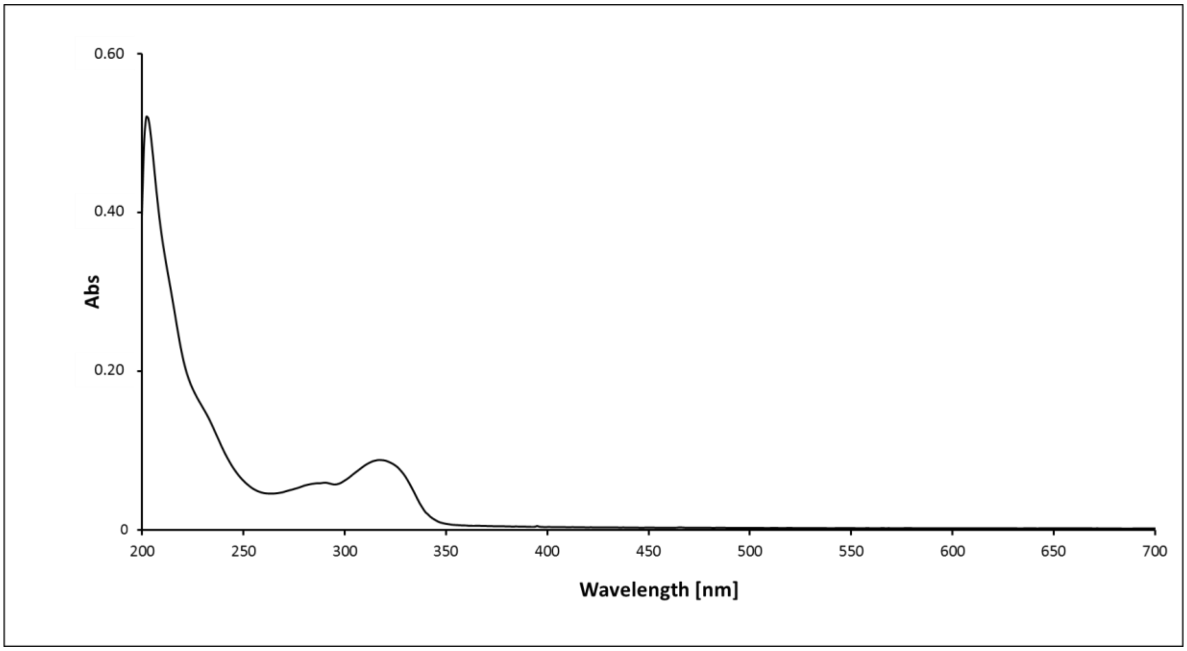

Figure S17: UV spectrum of compound **2** in MeOH.

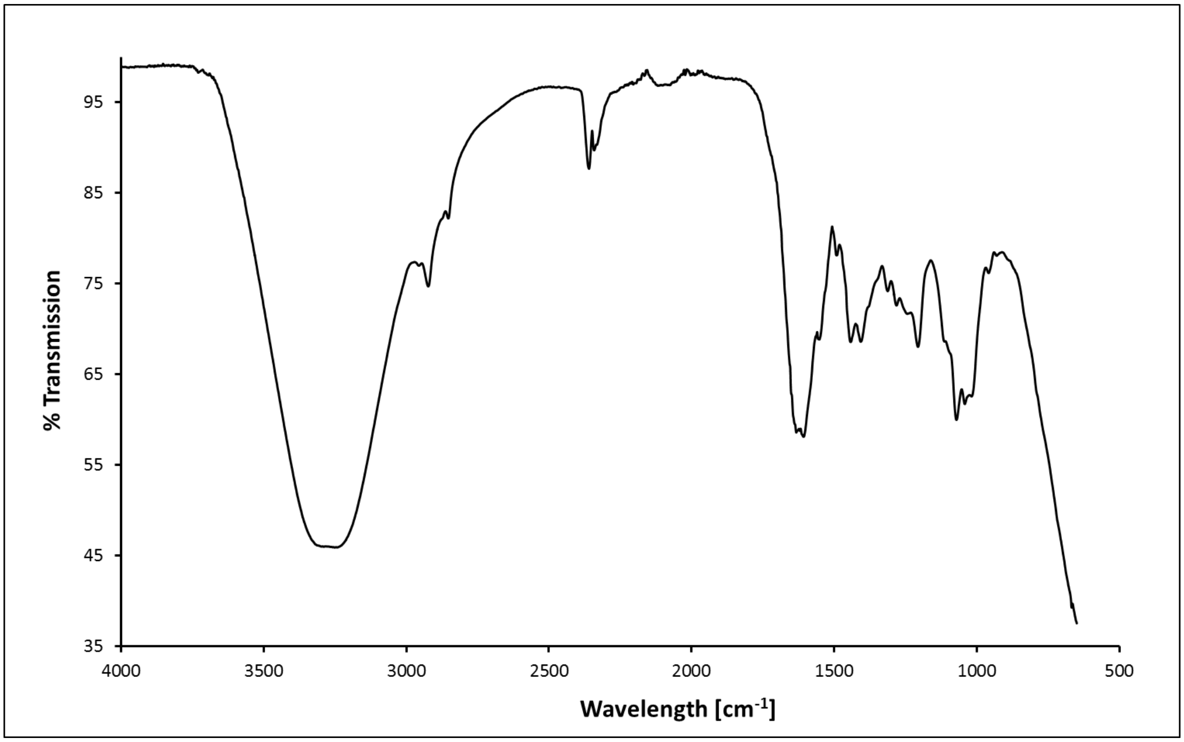

Figure S18: IR spectrum of compound **2** in MeOH.

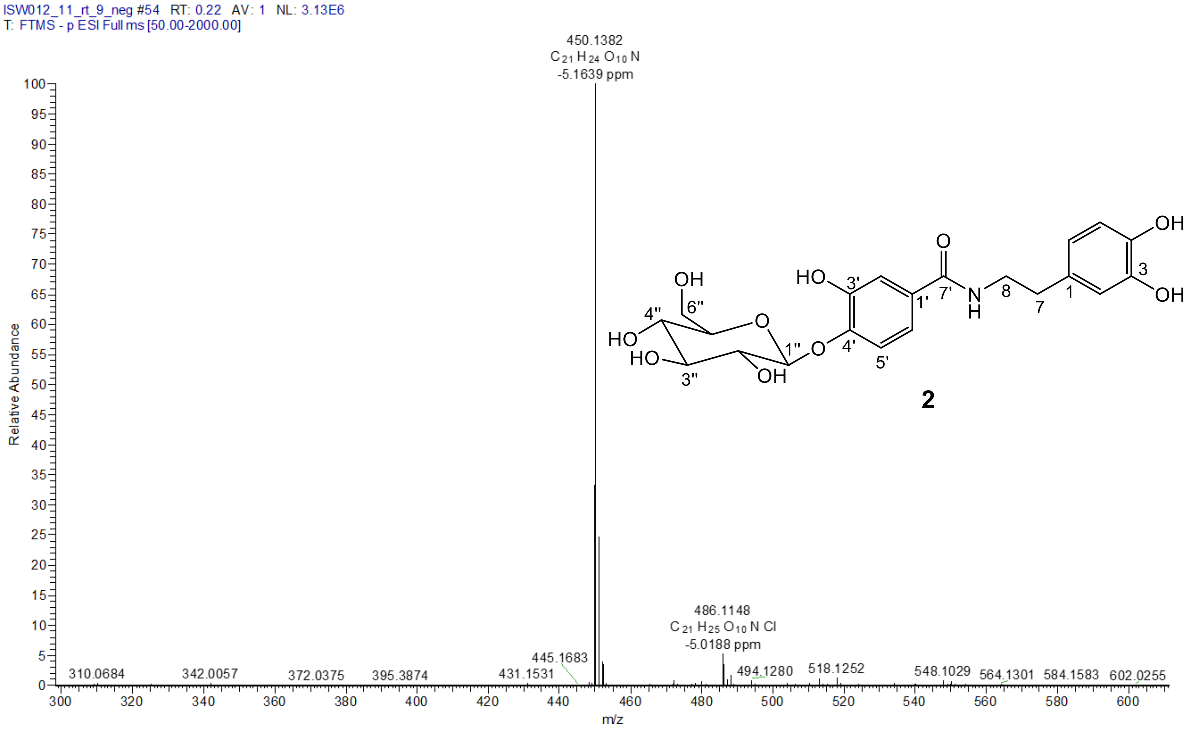

Figure S19: ESI-HRMS spectrum of compound **2** in negative ion mode.

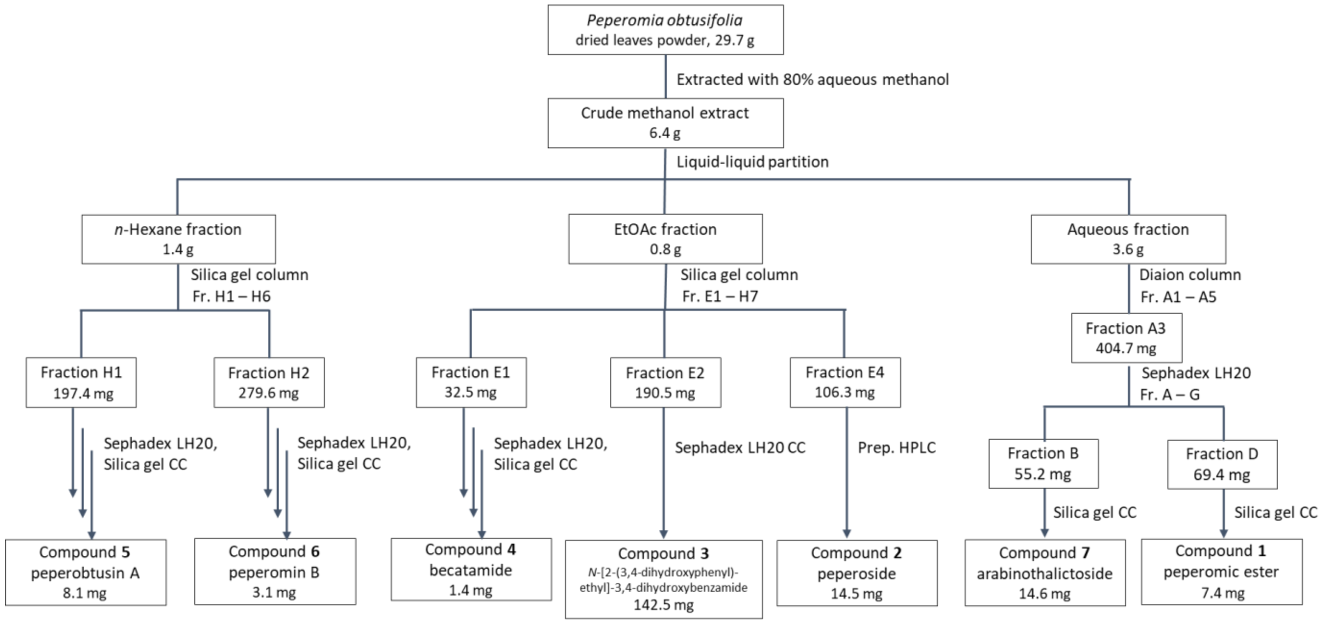

Figure S20: Isolation scheme for compounds **1**-**7**.
